# Estimates of burden and consequences of infants born small for gestational age in low and middle income countries with INTERGROWTH-21^st^ standard: analysis of CHERG datasets

**DOI:** 10.1136/bmj.j3677

**Published:** 2017-08-17

**Authors:** Anne CC Lee, Naoko Kozuki, Simon Cousens, Gretchen A Stevens, Hannah Blencowe, Mariangela F Silveira, Ayesha Sania, Heather E Rosen, Christentze Schmiegelow, Linda S Adair, Abdullah H Baqui, Fernando C Barros, Zulfiqar A Bhutta, Laura E Caulfield, Parul Christian, Siân E Clarke, Wafaie Fawzi, Rogelio Gonzalez, Jean Humphrey, Lieven Huybregts, Simon Kariuki, Patrick Kolsteren, John Lusingu, Dharma Manandhar, Aroonsri Mongkolchati, Luke C Mullany, Richard Ndyomugyenyi, Jyh Kae Nien, Dominique Roberfroid, Naomi Saville, Dianne J Terlouw, James M Tielsch, Cesar G Victora, Sithembiso C Velaphi, Deborah Watson-Jones, Barbara A Willey, Majid Ezzati, Joy E Lawn, Robert E Black, Joanne Katz

**Affiliations:** 1 Department of Pediatric Newborn Medicine, Brigham and Women’s Hospital, 75 Francis Street, Boston, MA 02115, USA; 2 Harvard Medical School, 25 Shattuck St, Boston, MA 02115, USA; 3 International Rescue Committee, 1730 M Street NW, Suite 505, Washington, DC 20036, USA; 4 Department of International Health, Johns Hopkins Bloomberg School of Public Health, 615 N. Wolfe St., Baltimore, MD 21205, USA; 5 Facuty of Epidemiology and Population Health, London School of Hygiene and Tropical Medicine, London WC1E 7HT, UK; 6 Maternal, Adolescent, Reproductive, and Child Health (MARCH) Centre, London School of Hygiene and Tropical Medicine, London WC1E 7HT, UK; 7 Department of Information, Evidence and Research, World Health Organization (WHO), Geneva, Switzerland, CH-1211; 8 Programa de Pós-graduacao em Epidemiologia, Universidade Federal de Pelotas, Rua Marechal Deodoro 1160, 30 piso, Centro, CEP 96020-220, Pelotas, RS, Brazil; 9 Mailman School of Public Health, Columbia University, 722 W 168th St, New York, NY 10032; 10 Centre for Medical Parasitology, Department of Immunology and Microbiology, University of Copenhagen, Oester Farimagsgade 5, 1014 Copenhagen K, Denmark; 11 Department of Nutrition, Gillings School of Global Public Health, University of North Carolina at Chapel Hill, 135 Dauer Drive, Chapel Hill, NC 27599, USA; 12 Carolina Population Center, University of North Carolina at Chapel Hill, 137 E. Franklin, Chapel Hill, NC 27516, USA; 13 Programa de Pós-graduação em Saúde e Comportamento, Universidade Católica de Pelotas, Félix da Cunha, 412, CEP 96010-000, Centro, Pelotas, RS, Brazil; 14 Center for Global Child Health, Hospital for Sick Children, 686 Bay Street, Toronto, ON, M5G A04, Canada; 15 Centre of Excellence in Women and Child Health, Aga Khan University, Stadium Road PO Box 3500, Karachi 74800, India; 16 Center for Human Nutrition, Johns Hopkins Bloomberg School of Public Health, 615 N. Wolfe Street, W2041, Baltimore, MD 21205 USA; 17 Women’s Nutrition, Bill and Melinda Gates Foundation, Seattle, WA 98102, USA; 18 Faculty of Infectious Disease and Tropical Diseases, London School of Hygiene and Tropical Medicine, London WC1E 7HT, UK; 19 Malaria Centre, London School of Hygiene and Tropical Medicine, London WC1E 7HT, UK; 20 Department of Global Health and Population, Harvard School of Public Health, 677 Huntington Ave, Boston, MA 02115, USA; 21 Department of Nutrition, Harvard School of Public Health, 677 Huntington Ave, Boston, MA 02115, USA; 22 Department of Epidemiology, Harvard School of Public Health, 677 Huntington Ave, Boston, MA 02115, USA; 23 Pontificia Universidad Católica de Chile, School of Medicine, Avenida Libertador General Bernardo O’Higgins #340, Santiago, Chile; 24 Clínica Santa María, Avenida Santa María 0410 Providencia, Santiago, Chile; 25 Zvitambo Institute for Maternal and Child Health Research, 16 Lauchlan Road, Meyrick Park, Harare, Zimbabwe; 26 Department of Food Safety and Food Quality, Ghent University, Coupure Links 653 – 9000 Ghent, Belgium; 27 Poverty, Health and Nutrition Division, International Food Policy Research Institute, 2033 K St, NW Washington, DC 20006-1002, USA; 28 Kenya Medical Research Institute, Centre for Global Health Research, PO Box 1578-40100, Kisumu, Kenya; 29 Centers for Disease Control and Prevention Kenya, Off Kisumu-Busia Highway, PO Box 1578-40100, Kisumu, Kenya; 30 National Institute for Medical Research, PO Box 5004, Tanga, Tanzania; 31 University of Copenhagen, Denmark; 32 Mother and Infant Research Activities (MIRA), YB Bhawan, Thapathali, Kathmandu 921, Nepal; 33 ASEAN Institute for Health Development, Mahidol University, 999 Phuttamonthon 4 Rd, Salaya, Nakhon Pathom 73170, Thailand; 34 Vector Control Division, Ministry of Health, Uganda, Plot 6 Lourdel Rd, Nakasero, Kampala, Uganda; 35 Fetal Maternal Medicine Unit, Clinica Davila, Avenida Recoleta 464, Santiago, Chile; 36 Faculty of Medicine, Universidad de Los Andes, Avda San Carlos De Apoquindo 2200, Santiago, Chile; 37 Belgian Health Care Knowledge Centre, Boulevard du Jardin Botanique 55, Brussels, Belgium; 38 Institute for Global Health, University College London Institute of Child Health, London WC1N 1EH, UK; 39 Department of Clinical Sciences, Liverpool School of Tropical Medicine, Liverpool, L3 5QA, UK; 40 Malawi-Liverpool-Wellcome Trust Clinical Research Programme, College of Medicine, University of Malawi, PO Box 30096, Chichiri, Blantyre 3, Malawi; 41 Department of Global Health, Milken Institute School of Public Health, George Washington University, 950 New Hampshire Ave, NW, Suite 400, Washington, DC 20052, USA; 42 Department of Paediatrics, Chris Hani Baragwaneth Hospital, Faculty of Health Sciences, University of Witwatersrand, Soweto, Johannesburg, South Africa; 43 Clinical Research Department, London School of Hygiene and Tropical Medicine, London, UK; 44 Mwanza Intervention Trial Unit, National Institute for Medical Research, Mwanza, Tanzania; 45 MRC-PHE Centre for Environment and Health, Department of Epidemiology and Biostatistics, School of Public Health, Imperial College, London, London W2 1PG, UK; 46 Institute for International Programs, Johns Hopkins Bloomberg School of Public Health, Johns Hopkins University, Baltimore, MD, USA

## Abstract

**Objectives** To estimate small for gestational age birth prevalence and attributable neonatal mortality in low and middle income countries with the INTERGROWTH-21^st^ birth weight standard.

**Design** Secondary analysis of data from the Child Health Epidemiology Reference Group (CHERG), including 14 birth cohorts with gestational age, birth weight, and neonatal follow-up. Small for gestational age was defined as infants weighing less than the 10th centile birth weight for gestational age and sex with the multiethnic, INTERGROWTH-21^st^ birth weight standard. Prevalence of small for gestational age and neonatal mortality risk ratios were calculated and pooled among these datasets at the regional level. With available national level data, prevalence of small for gestational age and population attributable fractions of neonatal mortality attributable to small for gestational age were estimated.

**Setting** CHERG birth cohorts from 14 population based sites in low and middle income countries.

**Main outcome measures** In low and middle income countries in the year 2012, the number and proportion of infants born small for gestational age; number and proportion of neonatal deaths attributable to small for gestational age; the number and proportion of neonatal deaths that could be prevented by reducing the prevalence of small for gestational age to 10%.

**Results** In 2012, an estimated 23.3 million infants (uncertainty range 17.6 to 31.9; 19.3% of live births) were born small for gestational age in low and middle income countries. Among these, 11.2 million (0.8 to 15.8) were term and not low birth weight (≥2500 g), 10.7 million (7.6 to 15.0) were term and low birth weight (<2500 g) and 1.5 million (0.9 to 2.6) were preterm. In low and middle income countries, an estimated 606 500 (495 000 to 773 000) neonatal deaths were attributable to infants born small for gestational age, 21.9% of all neonatal deaths. The largest burden was in South Asia, where the prevalence was the highest (34%); about 26% of neonatal deaths were attributable to infants born small for gestational age. Reduction of the prevalence of small for gestational age from 19.3% to 10.0% in these countries could reduce neonatal deaths by 9.2% (254 600 neonatal deaths; 164 800 to 449 700).

**Conclusions** In low and middle income countries, about one in five infants are born small for gestational age, and one in four neonatal deaths are among such infants. Increased efforts are required to improve the quality of care for and survival of these high risk infants in low and middle income countries

## Introduction

Neonatal conditions are responsible for an increasing proportion of deaths of children aged under 5 and are a key focus of the post-2015 development agenda and the Every Newborn Action Plan.^1^ ^2^ Preterm birth, intrapartum related events, and neonatal infections are the main direct causes of neonatal mortality.^3^ Other risk factors for mortality, however, such as intrauterine growth restriction, are not classified as underlying or immediate causes of death according to ICD (international classification of diseases) rules.^4^ To most effectively target interventions to accelerate reductions in neonatal mortality, it is critical to quantify the attributable burden of mortality from major neonatal risk factors that are not classified as underlying or direct causes of death.

Infants born small for gestational age are defined by the WHO Expert Committee^5^ and the American College of Obstetrics and Gynecology^6^ as those weighing below the 10th centile of birth weight by sex for a specific completed gestational age of a given reference population. It is commonly used as a proxy for intrauterine growth restriction and, in settings with a high prevalence of small for gestational age, is more likely to be because of fetal intrauterine growth restriction.^7^

Infants born small for gestational age carry a considerably higher risk of mortality and morbidity in the neonatal period and beyond. They are more likely to have neonatal infections, perinatal respiratory depression, jaundice, polycythemia, hypoglycemia, poor feeding, and hypothermia. These morbidities in turn place them at higher risk of death. In a pooled analysis by the Child Health Epidemiology Reference Group (CHERG), small for gestational age was associated with increased risk of neonatal and postneonatal mortality (risk ratio 1.83 (95% confidence interval 1.34 to 2.50) for neonatal mortality; 1.90 (1.32 to 2.73) for postneonatal infant mortality) compared with infants born at an appropriate size for gestational age (≥10% birth weight for gestational age). The risk was even higher among those born both preterm and small for gestational age.^8^ Infants born small for gestational age also have an increased risk of delayed neurodevelopment and poor linear growth,^9^ with those born term and preterm being 2.4 and 4.5 times, respectively, more likely to be stunted in childhood than term babies infants born appropriate size for gestational age.^10^ Modifiable risk factors for small for gestational age include poor maternal nutrition,^11^ maternal infections and other morbidities,^12^ young maternal age,^13^ and short birth spacing.^14^ Intrauterine programming and genetic modulation have also been postulated as mechanisms resulting in small for gestational age and increased risk of morbidity later in life, predisposing those infants to higher risk of insulin resistance, obesity, dyslipidaemia, and hypertension in adulthood.^15^ ^16^

Epidemiologic estimates of small for gestational age vary substantially based on the reference population.^17^ The use of a single universal growth standard versus local/national ethnic specific growth references is still heavily debated.^18^ While genetic potential for growth might differ across populations,^19^ ^20^ this contribution is believed by some to play a smaller role in low and middle income countries on infant size at birth, compared with the impact of maternal undernutrition and pregnancy morbidity.^5^ As part of the CHERG, we investigated the global burden of infants born small for gestational age, the contribution of pregnancy risks, and the potential impact of preventive interventions to deal with risk factors to optimize fetal growth globally. The INTERGROWTH-21^st^ project^21^ (henceforth referred to as Intergrowth) established the first international, multiethnic standard including well dated pregnancies from eight geographically defined populations, and enables a common single standard to describe optimal and aspirational fetal growth around the world.^21^ The Intergrowth study found that among healthy pregnant women with adequate nutrition, fetal growth was comparable across different populations around the world.^22^ Therefore, Intergrowth was chosen as the standard for this analysis in a prescriptive sense, to describe the global burden of suboptimal fetal growth. Choice of unselected, local, national population references would simply describe fetal growth with current rates of exposure to undernutrition and pregnancy morbidities in low and middle income countries, and would not allow us to target the optimization of fetal growth globally from a population health perspective.

The population attributable fraction reflects the burden of disease attributable to a causal risk factor if it were reduced from the current exposure level to a theoretical minimum counterfactual distribution. This allows the estimation of the potential reduction in mortality if the exposures were eliminated or, in other words, the attribution of indirect deaths caused by given risk factors. We estimated the number of infants born small for gestational age in low and middle income countries in 2012 using the new Intergrowth standard, the number of neonatal deaths attributable to being small for gestational age in these countries, and the number of neonatal deaths that could be prevented by reducing the prevalence of small for gestational age to a theoretical minimum level of 10% in these countries. We also compared our results to previously published estimates of prevalence of small for gestational aged derived from a US birth weight reference.^23^

## Methods

We have built on prior analyses of the CHERG on the burden and risk of small for gestational age and preterm birth in low and middle income countries.^8^ ^24^ ^25^ An investigator group (CHERG SGA-Preterm Birth Working Group) was established in 2009 to conduct a set of analyses related to small for gestational age and preterm birth. To identify datasets to include in the analyses, we reviewed Medline, WHO regional databases (African Index Medicus, LILAS, EMRO), manuscript bibliographies, and grey literature to identify cohorts from low and middle income countries with information on gestational age and birth weight and that systematically recorded vital status until 28 days of life. We included datasets that were population based, representing most deliveries from certain geographic or catchment areas, whether home or facility based. We excluded datasets with more than 25% missing data on birth weight, gestational age, or neonatal follow-up; measured weight only after 72 hours of life; did not have systematic follow-up of vital status in the first month of life; or had imprecise gestational age data (determined in whole months or deemed “inaccurate” by study investigators). Full details on the literature review process and selection of datasets have been previously published by our research group.^8^ Principal investigators were requested to either individually conduct analyses or to share their data with the working group. We included 14 birth cohorts in this analysis.^26^ ^27^ ^28^ ^29^ ^30^ ^31^ ^32^ ^33^ ^34^ ^35^ ^36^ ^37^ ^38^ ^39^ The original studies were from prospective studies, including longitudinal birth cohorts (n=4) and pregnancy/neonatal intervention trials (n=10). The original datasets and study descriptions are shown in appendix 1. Most datasets were from 2000 onwards, with three studies from the 1990s. This analysis excludes datasets used in previous CHERG analyses that were from before 1990 or were not directly available to the analysts for this study.^8^

In previously published analyses, we estimated the prevalence of small for gestational age and preterm birth in low and middle income countries for the year 2010, using the US 1991 birth population as a reference (US National Center for Health Statistics, n=3 808 689 live births in 1991)^25^ and the associations of small for gestational age (defined using this US reference) and preterm birth with both neonatal and postneonatal infant mortality, respectively.^8^ In the current analyses, we estimate the numbers of small for gestational age births and neonatal deaths attributable to this in low and middle income countries in 2012 using the international Intergrowth birth weight standard to define small for gestational age.

### Exposure definitions

Small for gestational age was defined as a birth weight less than the 10th centile for a specific completed gestational age by sex, using the Intergrowth standard.^21^ ^40^ The Intergrowth cutoffs were taken from two publications, the first for gestational age ≥33 weeks^21^ and the second for <33 completed weeks of gestation.^40^ In the CHERG cohorts, gestational age was estimated with ultrasonography or best obstetric estimate including ultrasonography in six studies, date of last menstrual period alone for five studies, postnatal clinical exam (Ballard and Capurro) for two studies, and a combination in one study (see appendix 1 for more details). We also compared these results with small for gestational age defined by the 1991 US national population reference, which was used in the original CHERG analysis.^23^ Preterm birth was defined as gestational age of <37 completed weeks. Low birth weight was defined as birth weight of <2500 g. We created four mutually exclusive exposures: term appropriate for gestational age (as reference), term small for gestational age, preterm appropriate for gestational age, and preterm small for gestational age (fig 1). Term small for gestational age was further separated into low birth weight and not low birth weight to differentiate the mortality burden associated with smaller and larger term small for gestational age infants.

**Figure f1:**
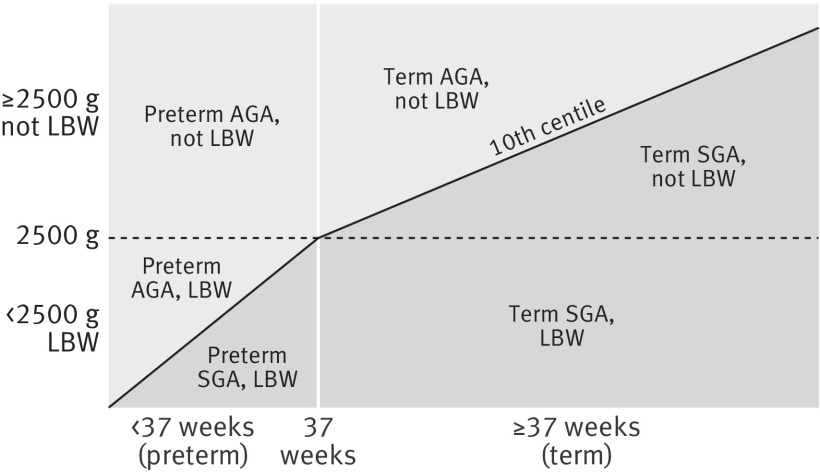
**Fig 1** Combinations of exposure categories of preterm birth, small for gestational age (SGA), and low birth weight (LBW, <2500 g). AGA=appropriate for gestational age

### Population distribution of small for gestational age and preterm birth

CHERG previously estimated national and regional prevalences of preterm birth^24^ and small for gestational age for 2010^25^ and 2012^41^ using the US 1991 population birth weight reference.^23^ To estimate prevalence of small for gestational age with the Intergrowth standard,^21^ we calculated the percentage change of term and preterm small for gestational age births in the 14 CHERG datasets, comparing the US population reference with the Intergrowth standard. We performed meta-analysis using random effects to pool the percentage change at the regional level (Asia, Africa, Americas) and multiplied the region specific adjustment factor by the previously estimated national level prevalences of small for gestational age from 2012 (see appendix 2).^42^ We performed a similar process to calculate the proportion of term small for gestational age infants with low birth weight.

### Risk ratios for neonatal mortality

Mortality was analyzed in the neonatal (birth-28 days of life) period. For each dataset, we calculated risk ratios for neonatal mortality (death within first 28 days of life) for preterm birth and small for gestational age, classified using the US 1991 reference and Intergrowth standard.^8^ We pooled risk ratios for neonatal mortality for categories of small for gestational age separately for each reference/standard, at the regional level, with random effects meta-analysis to calculate DerSimonian-Laird pooled risk ratios and 95% confidence intervals (see appendix 3).

### Neonatal deaths attributable to small for gestational age birth

We used neonatal mortality rates from the Inter-Agency Group for Child Mortality Estimation^43^ and live births from the UN Population Division^44^ for the year 2012.

Population attributable fraction, the “proportion of cases for an outcome of interest that can be attributed to a given risk factor,” is defined as the (incidence rate in population−incidence rate in unexposed)/incidence rate in the total population.^45^ In this paper, we estimated the neonatal deaths that would result if the causally related risk factor (in this case, small for gestational age) was reduced from its current exposure level to a theoretical minimum counterfactual distribution. Using established methods of comparative risk assessment^46^ used for the global burden of disease,^47^ we estimated the total number of neonatal deaths that were attributable to small for gestational age, as well as the number of such deaths that would be prevented if the prevalence of small for gestational age was reduced to a theoretical minimum level of 10% in all low and middle income countries, the distribution expected among low risk mothers, similar to the Intergrowth population. For the co-occurrence of preterm and small for gestational age, the theoretical minimum risk level of mortality was that of preterm appropriate for gestational age infants to attribute the deaths related only to small for gestational age. The population attributable fraction for multiple category exposures can be estimated by the equation for potential impact fraction (fig 2).^45^ ^48^ ^49^ We calculated population attributable fractions and number of neonatal deaths averted at the national level and then aggregated by UN-Millennium Development Goal regions for 138 low and middle income countries.

**Figure f2:**
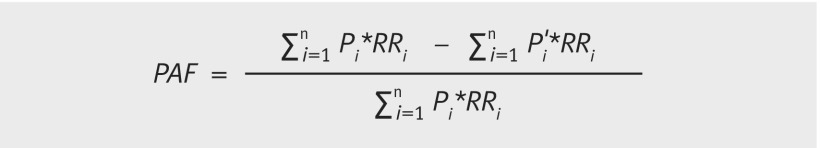
**Fig 2** Equation for population attributable fraction (PAF). Pi=proportion of population at exposure level i, current exposure; P'i = proportion of population at exposure level i, counterfactual or ideal level of exposure; RR= risk ratio at exposure level i; n=number of exposure levels

### Population attributable fraction uncertainty estimates

Methods to estimate uncertainty ranges have been developed by the CHERG by using a bootstrap approach as opposed to jackknife procedures to allow more plausible uncertainty ranges.^3^ These methods are described in detail in appendix 4.

### Patient involvement

This study was a secondary data analysis of existing datasets, which did not involve new direct contact with patients. For all parts of these secondary data analyses, patients, caregivers, and lay people were not involved in the development of the research question, study design, or outcome measures, nor the interpretation or writing up of the results. Some of the original studies contributing to this analysis included recruitment of participants by lay community health workers. Data from this study will be published and made publicly available. Investigators might share the results with local ministries of health, patients (including original study participants), and relevant medical organizations in the respective countries where the original studies were conducted.

## Results

### Small for gestational age live births in low and middle income countries in 2012

Table 1 and figure 3 show the estimated numbers and prevalence of small for gestational age among live births for the year 2012, defined by the Intergrowth standard. National level estimates are available in appendix 5. The regional numbers are compared with estimates using the US 1991 population reference in appendix 6. In low and middle income countries, 23.3 million infants were born small for gestational age as defined by the Intergrowth standard, with a prevalence of 19.3%. The highest number and prevalence of such births was in South Asia, at 12.5 million (34.2%) infants. Most (62.7%) small for gestational age births occurred in South or South East Asia. An estimated 5.6 million infants born in sub-Saharan Africa were small for gestational age (prevalence 16.5%).

**Table 1 tbl1:** Numbers of 1000s of infants born small for gestational age (SGA) in 2012 with INTERGROWTH-21^st^ birth weight standard in low and middle income countries in regions covered by UN Millennium Development Goals

	No of live births (1000s)	No (UR^*^) of term SGA (1000s)		No (UR^*^) of preterm SGA (1000s	Total No (UR^*^) of SGA (1000s)	% prevalence (UR^*^) SGA
		Not low birth weight	Low birth weight^†^			
Caucasus/Central Asia	1774.3	87.0 (49.2 to 148.6)	89.1 (50.8 to 152.0)	19.4 (9.4 to 41.6)	195.5 (121.1 to 314.1)	11.0 (6.2 to 19.3)
Eastern Asia	19 097.2	387.4 (170.4 to 788.5)	396.8 (180.0 to 799.2)	165.4 (82.6 to 320.1)	949.5 (536.7 to 1735.4)	5.0 (2.4 to 10.4)
Latin America/Caribbean	10 833.3	516.3 (406.5 to 1157.1)	303.2 (241.1 to 687.5)	110.8 (83.3 to 243.5)	930.3 (793.2 to 2019.5)	8.6 (6.7 to 19.3)
Northern Africa	3989.8	120.9 (59.8 to 233.0)	102.6 (49.8 to 200.6)	24.6 (10.3 to 53.7)	248.2 (138.9 to 455.7)	6.2 (3.0 to 12.2)
Oceania	266.4	20.0 (12.1 to 31.6)	20.4 (12.9 to 32.4)	2.3 (1.0 to 6.3)	42.7 (28.1 to 66.0)	16.0 (9.8 to 26.4)
South East Asia	9691.1	941.7 (587.6 to 1448.6)	964.6 (609.8 to 1499.1)	183.5 (88.0 to 386.4)	2089.9 (1403.2 to 3157.7)	21.6 (14.2 to 37.7)
South Asia	36 625.8	5908.5 (3849.1 to 8672.5)	6052.1 (3974.6 to 8954.2)	577.1 (291.6 to 1162.3)	12 537.7 (8651.8 to 18 100.0)	34.2 (22.2 to 51.3)
Sub-Saharan Africa	33 727.5	2829.5 (1522.8 to 5105.4)	2400.6 (1253.1 to 4297.7)	345.0 (158.9 to 716.8)	5575.2 (3276.3 to 9277.2)	16.5 (8.7 to 25.1)
Western Asia	4 844.9	346.0 (213.3 to 538.3)	354.4 (224.7 to 558.5)	56.2 (28.2 to 118.2)	756.6 (504.6 to 1154.3)	15.6 (9.6 to 25.1)
Total	120 850.2	11 157.4 (8195.4 to 15 798.3)	10 683.9 (7616.9 to 15 017.0)	1484.3 (902.2 to 2628.7)	23 325.6 (17 599.3 to 31 914.8)	19.3 (11.9 to 32.1)

*Uncertainty range (UR) derived with bootstrap approach (see appendix 4).

†≤2500 g.

**Figure f3:**
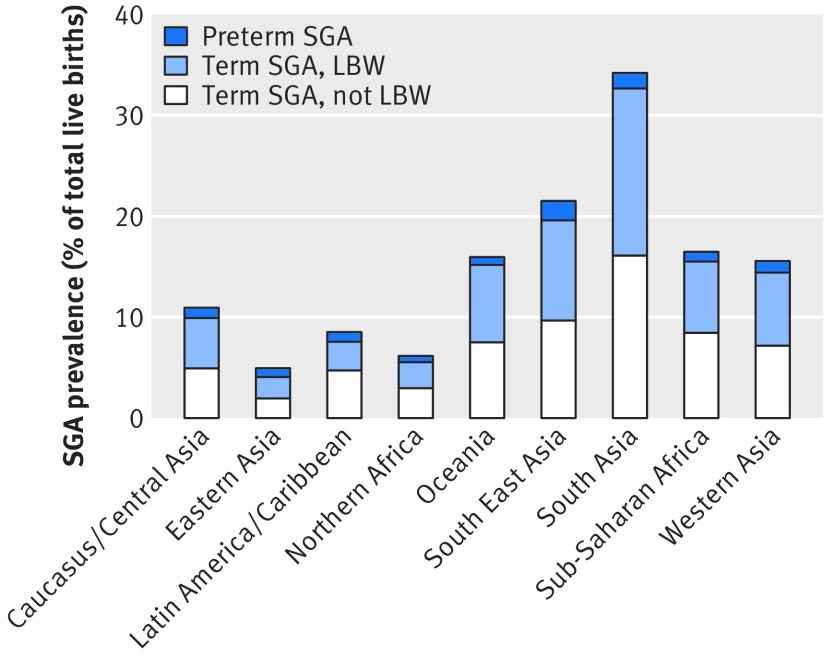
**Fig 3** Prevalence of infants born small for gestational age (SGA) among live births in low and middle income countries in 2012, by UN-MDG region. LBW=low birth weight (<2500 g)

The total number of estimated small for gestational age births was 27% lower when we used the Intergrowth standard compared with the US population reference (appendix 6). The largest absolute difference was in the number of term small for gestational age infants who weighed above 2500 g (18.8 million by US 1991 reference compared with 11.2 million with the Intergrowth standard, 41% reduction). The greatest relative reduction was among the preterm infants (47% lower with Intergrowth).

### Neonatal deaths attributable to small for gestational age in 2012

An estimated 606 500 neonatal deaths (21.9% of neonatal deaths) in low and middle income countries were attributable to being born small for gestational age in 2012, as defined with the Intergrowth standard (table 2, fig 4). In total, 410 600 were among term small for gestational age infants, the majority weighing <2500 g, and an estimated 195 900 neonatal deaths among preterm small for gestational age infants. The largest number of neonatal deaths attributable to small for gestational age was in South Asia, where the prevalence of small for gestational age was highest (at 34%) and 26% of neonatal deaths were attributed to this risk factor. About half of the neonatal deaths attributable to small for gestational age (322 700) occurred in South or South East Asia. National level estimates are shown in appendix 7.

**Table 2 tbl2:** Numbers of 1000s of neonatal deaths in 2012 attributable to term and preterm infants born small for gestational age (SGA) in low and middle income countries in regions covered by UN Millennium Development Goals

	No of live births (1000s)^*^	No (UR^*^) of neonatal deaths (1000s)					Population attributable fraction^†^ (UR^*^)
		Total (1000s)	Term SGA		Preterm SGA	All SGA	
			Not low birth weight	Low birth weight			
Caucasus/Central Asia	1774.3	26.5	—	1.5 (0.9 to 2.5)	2.3 (1.2 to 4.3)	3.8 (2.6 to 5.8)	14.3 (9.9 to 22.0)
Eastern Asia	19 097.2	158.9	—	4.2 (1.9 to 8.3)	11.9 (6.3 to 21.5)	16.1 (10.3 to 25.9)	10.1 (6.5 to 16.3)
Latin America/Caribbean	10 833.3	105.9	—	8.6 (6.5 to 17.5)	18.7 (13.1 to 29.6)	27.3 (24.8 to 40.4)	25.8 (23.4 to 38.1)
Northern Africa	3989.8	50.6	1.6 (0.8 to 2.9)	2.7 (1.4 to 5.0)	0.9 (0.4 to 1.8)	5.2 (3.1 to 8.8)	10.3 (6.1 to 17.3)
Oceania	266.4	5.7	—	0.5 (0.3 to 0.8)	0.4 (0.2 to 0.9)	0.9 (0.7 to 1.4)	15.8 (11.4 to 24.0)
South East Asia	9691.1	143.9	—	14.5 (9.2 to 21.9)	18.5 (9.5 to 33.4)	33.0 (24.4 to 47.3)	22.9 (17.0 to 32.9)
South Asia	36 625.8	1127.3	—	175.8 (123.0 to 248.1)	113.9 (63.2 to 204.4)	289.7 (227.6 to 383.1)	25.7 (20.2 to 34.0)
Sub-Saharan Africa	33 727.5	1090.2	72.2 (41.7 to 118.3)	123.2 (68.7 to 198.5)	23.9 (11.4 to 47.3)	219.3 (143.3 to 325.4)	20.1 (13.1 to 29.8)
Western Asia	4844.9	63.4	—	5.6 (3.6 to 8.4)	5.4 (2.8 to 10.0)	11.0 (8.2 to 15.5)	17.4 (13.0 to 24.5)
Total	120 850.2	2772.4	73.8 (42.5 to 120.7)	336.8 (250.7 to 453.8)	195.9 (123.0 to 325.0)	606.5 (494.8 to 772.9)	21.9 (17.8 to 27.9)

*Uncertainty range (UR) derived with bootstrap approach (see appendix 4).

†%of neonatal deaths attributable to SGA.

**Figure f4:**
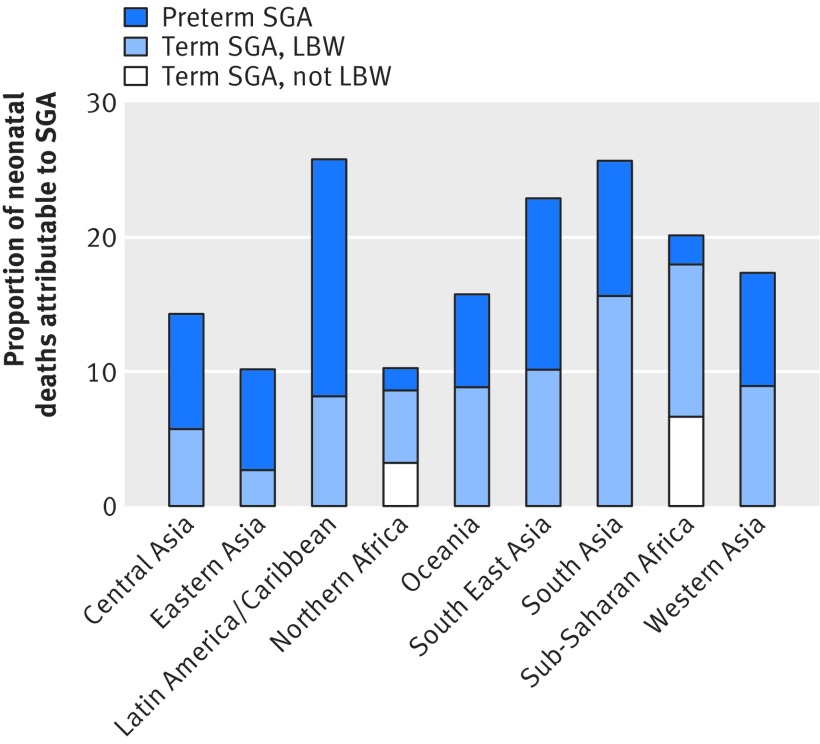
**Fig 4** Proportion of total neonatal deaths attributable to infants born small for gestational age (SGA) in low and middle income countries in 2012 by UN-MDG region. LBW=low birth weight (<2500 g)

Compared with the US 1991 birth population, the total number of neonatal deaths attributable to small for gestational age with the Intergrowth standard was about 21% lower (appendix 8). There was also a relatively large reduction (48%) in the number of neonatal deaths attributed to small for gestational age in term infants who weighed ≥2500 g as well as preterm small for gestational age (42%) infants with the Intergrowth classification, in large part because of the lower prevalence of births in these groups with the Intergrowth standard.

Table 3 lists the 10 countries with the largest numbers of estimated deaths in infants born small for gestational age in 2012. The highest number was in India, where 9.1 million such infants (36.5% of live births) were born in 2012, with 202 300 attributable neonatal deaths. Pakistan and Nigeria also had high numbers of both infants born small for gestational age (1.7 million in Pakistan, 1.1 million in Nigeria)^25^ and attributable neonatal deaths (53 700 and 51 800, respectively). The highest proportions of neonatal deaths that were attributable to small for gestational age were in Sudan (28.7%), Pakistan (26.5%), and India (26.0%).

**Table 3 tbl3:** Ten countries with highest burden of neonatal mortality attributable to infants born small for gestational age (SGA)

	No of live births (1000s)	Neonatal mortality rate^*^ 2012	Preterm birth rate 2012 (%)	Prevalence of SGA (%)	No of attributable neonatal deaths (1000s)			Population attributable fraction^†^
					Term SGA	Preterm SGA	All SGA	
1 India	25 000	30.9	13.1	36.5	126.3	76	202.3	26.0
2 Pakistan	4800	42.2	15.8	36.0	30.8	22.9	53.7	26.5
3 Nigeria	6800	39.2	12.2	15.6	45.8	6.0	51.8	19.4
4 Bangladesh	3100	24.4	14.1	30.5	10.3	8.1	18.3	24.2
5 China	19 000	8.5	6.9	4.6	3.8	11.3	15.1	9.6
6 Indonesia	4800	15.0	15.6	18.0	5.7	8.8	14.5	19.9
7 Ethiopia	3000	29.0	10.2	21.4	20.8	1.6	22.4	25.5
8 Philippines	2300	14.0	14.9	25.6	3.6	3.7	7.3	22.7
9 DR Congo	2700	43.5	12.0	14.5	19.0	2.6	21.7	18.3
10 Sudan	1200	28.6	13.4	28.0	9.3	0.7	10.0	28.7

*Per 1000 live births.

†% of neonatal deaths attributable to SGA.

In an international, multiethnic setting of optimal nutrition and health in pregnancy, we would expect 10% of infants to be born small for gestational age. Reduction in the prevalence of small for gestational age from 19.3% to 10.0% in low and middle income countries could reduce total neonatal deaths by 9.2% (254 600 neonatal deaths; uncertainty range 164 800 to 449 700, appendix 9). The highest impact on newborn lives saved would be seen in India (n=109 000), Pakistan (27 000), Nigeria (21 000), and Ethiopia (21 000).

## Discussion

Our study reports the first global estimates of the burden of small for gestational age among live births and the contribution of small for gestational age as a risk factor for (or indirect cause of) neonatal mortality with the Intergrowth standard, for the year 2012. We estimate that in low and middle income countries, 23.3 million infants (19.3%) were born small for gestational age (11.2 million term and not low birth weight, 10.7 million term and low birth weight, 1.5 million preterm), and about 606 500 neonatal deaths (21.9%) were attributable to being born small for gestational age. The highest burden was in South Asia, where up to 34% of infants might be born small for gestational age and 26% of neonatal deaths were attributable to small for gestational age. If the prevalence of small for gestational age were reduced to a level of 10% (the prevalence that would be expected in an international population of optimal nutrition and health in pregnancy) in all low and middle income countries, an estimated 9.2% of neonatal deaths (n=254 600) could be averted.

In this analysis, we used the Intergrowth standard to classify small for gestational age infants^21^ ^50^ because our primary objective was to determine the global burden of suboptimal fetal growth, aspiring to a scenario where all mothers’ nutritional and health needs are met. There is still extensive debate, however, about the use of a single universal standard versus ethnic specific or customized fetal growth standards, and whether Intergrowth’s population was too selective. While genetic potential for growth can differ across populations, we think that in low and middle income countries these differences play a smaller role compared with the much larger variability in maternal nutritional status and health in pregnancy.^5^ Intergrowth showed in their cohort (n=20 486) that fetuses in healthy well nourished pregnant women from eight different geographic regions grew similarly across diverse geographic regions.^21^ ^22^ Furthermore, the neonatal birth weights of Intergrowth are comparable with the WHO Child Growth Standards for term neonates.^21^ We therefore chose the Intergrowth standard as the most appropriate prescriptive standard to describe the global burden of suboptimal fetal growth and the population impact of public health interventions to deal with this.

There is also an argument, however, for the use of ethnic specific fetal growth references. In the Netherlands, Visser and colleagues published population birth weight reference curves and showed that Dutch Hindustani babies were systematically of lower birth weight than other ethnic groups, up to 300 g at certain gestational weeks.^51^ Two recent studies that used longitudinal ultrasound data in populations at low obstetric risk have shown ethnic differences in fetal growth, as measured by ultrasound estimated fetal weight.^19^ ^20^ The NICHD Fetal Growth study in the US included women at low obstetric risk and found significant differences in estimated fetal weight after 20 weeks, with lower weight among Hispanic, Asian, and Black women compared with non-Hispanic white women.^19^ The WHO multinational longitudinal study of ultrasound biometric measurements and estimated fetal weight included low risk pregnancies from 10 countries, and reported significant differences in estimated fetal weight and birth weight between countries, with the lowest median birth weight in India.^20^ There are differences that could explain the discrepant findings between these studies and Intergrowth. Intergrowth had stricter nutritional criteria such as excluding mothers with height <153 cm, whereas the NICHD and WHO did not exclude mothers based on height. These latter studies were also of smaller sample size, were primarily based on estimated fetal weight by ultrasound, and have not yet published birth weight standards. The use of individual level, customized birth weight standards that account for ethnicity and maternal characteristics (height, weight, parity) more accurately identified small for gestational age infants at risk of stillbirth or neonatal mortality in New Zealand.^52^ The application of customized charts, however, is not possible for population level estimates, and such charts also include targetable risk factors for small for gestational age in their growth predictions (for example, maternal undernutrition).

National or ethnic references are also often challenged by the fact that they are developed from the local population and are unselected, including all the pregnancies and existing morbidities of a local population/catchment area. Though these describe local patterns of fetal growth, they also include pregnancies that are affected by undernutrition and morbidities such as infections (for example, malaria, syphilis) and hypertension. Therefore, small for gestational age simply defines the lowest 10% of these populations, but this classification does not identify the many more newborns affected by poor growth. Use of local curves results in a predefined prevalence of small for gestational age close to 10%, irrespective of a population’s health and nutritional status. For example, with the Bhatia reference that was developed for single liveborn infants in India,^53^ the estimated number of infants born small for gestational age in South Asia for 2012 would be reduced to 3.8 million infants (10.5% of live births) in the year 2012,^17^ compared with 12.5 million (34%) with the Intergrowth standard.

Intergrowth’s 10th centile birth weight cutoff was generally 150-200 g lower across gestational weeks compared with the commonly cited US national reference population. For example, for boys born after 37 completed gestational weeks, the 10th centile was 2380 g (Intergrowth) versus 2596 g (US reference population).^54^ Use of the Intergrowth standard reduced the numbers of infants classified as small for gestational age by a relative 27% overall compared with the US 1991 reference, particularly term infants weighing ≥2500 g and late preterm (33-37 weeks). With a lower birth weight cut off, the Intergrowth standard identified a higher risk population.

The co-occurrence of prematurity and small for gestational age places infants at substantially, and potentially synergistically, higher risk of morbidity and neonatal mortality^54^ compared with their counterparts who experience prematurity or small for gestational age alone. Each year, an estimated 1.5 million infants are born both preterm and small for gestational age, and these small high risk infants are a top priority for public health interventions. The estimates related to infants born both preterm and small for gestational age were the most affected by the choice of growth standard. Prior population birth weight references, such as the US reference used in our study, have included all pregnancies. In comparison, the Intergrowth study excluded pregnancies in women with morbidities, meaning that preterm births caused by common maternal morbidities in US populations, such as obesity and gestational diabetes, were removed from the study population. This resulted in fewer preterm births and the captured preterm births represented a healthier subset. The Intergrowth standard had lower 10th centile birth weight cut offs, more so for lower gestational ages, thus our estimates for small for gestational age among preterm births were 47% lower with the Intergrowth standard than with the US reference.^8^

The primary prevention of intrauterine growth restriction in low and middle income countries is an important intervention target, particularly in South Asia, where prevalence is high. The causes of intrauterine growth restriction vary by setting. While nutritional deficiency is expected to be the largest contributor in low and middle income countries, there are other causal mechanisms, such as maternal infections, placental insufficiency, pregnancy morbidity, and environmental exposures that contribute in these settings. Further research on the country or region specific epidemiology and appropriate context specific solutions will be necessary to deal with primary prevention effectively. A more immediate goal is targeting the coverage and quality of interventions to manage morbidities of infants born small for gestational age, which will also benefit preterm infants. An estimated 80% of neonatal deaths occur in infants of small size (small for gestational age and/or preterm).^41^ Small for gestational age infants have an increased risk of perinatal respiratory depression^55^ from chronic uteroplacental insufficiency^56^ and postnatal infections from retarded development of the immune system.^57^ Interventions such as neonatal resuscitation, management of sepsis, chlorhexidine antisepsis of the umbilical cord, and early breastfeeding support could successfully target the reduction in mortality associated with small for gestational age. The effect of kangaroo mother care in term infants born small for gestational age also deserves further evaluation.

### Limitations

The CHERG datasets had several limitations. Several of the cohorts were community based studies with some missing birth weight data; however, we included only datasets with limited amounts of missing data and weight measured within 72 hours of birth, using strict a priori inclusion criteria reported elsewhere.^8^ Also, data were available only from select countries within regions and thus might not be representative of the entire region. We therefore used pooled regional risk ratios, aiming to increase generalizability of the estimations, and national level prevalence and mortality rates. Concerted efforts are needed to improve the coverage and quality of birth weight data in low and middle income countries, particularly in South Asia and sub-Saharan Africa, where over half of infants are never weighed at birth.^58^ Another limitation was the heterogeneity and quality of measures of gestational age. Ultrasound measures were not available in most studies, and the remaining studies used date of last menstrual period or clinical newborn assessment. Three studies, in which date of last menstrual period was collected during routine pregnancy surveillance, are likely to be more accurate.^59^ Dating of gestational age remains a large obstacle for research and programmatic projects in low and middle income countries. Accurate and feasible methods of gestational age dating will be necessary in the future in these countries to improve our epidemiologic understanding of the burden of preterm birth and small for gestational age. Finally, our analyses only represented the burden of SGA among live born babies. Intrauterine growth restriction is an important cause of stillbirth, and not represented in this analysis.

### Conclusions

We estimated that 23.3 million infants (19.3%) were born small for gestational age and 606 500 (21.9%) neonatal deaths were attributable to small for gestational age in low and middle income countries in 2012. The largest burden was in South Asia, where 34% of infants were born small for gestational age and 289 700 neonatal deaths were attributable to small for gestational age. Beyond the neonatal period, small for gestational age infants are at increased risk of experiencing later morbidity in childhood, including poor linear growth and chronic non-communicable disease in adulthood, a large yet unquantified burden. Overcoming implementation barriers and increasing coverage of proved interventions to prevent fetal growth restriction and improve survival of small infants are key priorities to reduce neonatal mortality in low and middle income countries.

**What is already known on this topic**

Infants born small for gestational age are at risk for neonatal mortality
Small for gestational age is highly prevalent in low and middle income countries, particularly in South Asia
Prior global estimates of small for gestational age have used the US 1991 live birth data as the birth weight reference

**What this study adds**

An estimated 23.3 million infants (uncertainty range 17.6 million to 31.9 million; 19.3% of live births) were born small for gestational age in low and middle income countries in 2012, with the INTERGROWTH-21^st^ standard, the first international, multiethnic birth weight standard, as reference
In low and middle income countries in 2012, 606 500 (21.9%) (495 000 to 773 000) neonatal deaths were attributable to small for gestational age
The highest burden was in South Asia, where the prevalence of small for gestational age was the highest (34%) and the population attributable fraction of attributable neonatal deaths was 26%

## Web Extra

Extra material supplied by the author

Appendix 1: Studies that contributed data

Appendix 2: Conversion of prevalence of small for gestational age from US 1991 reference to INTERGROWTH-21^st^ standard

Appendix 3: Pooled risk ratios for neonatal mortality in 14 CHERG datasets, with US 1991 reference and INTERGROWTH-21^st^ standard

Appendix 4: Methods of uncertainty estimation

Appendix 5: National level estimates of small for gestational age prevalence in 2012, INTERGROWTH-21^st^ standard

Appendix 6: Numbers of small for gestational age infants born in 2012 comparing INTERGROWTH-21^st^ birth weight standard with US 1991 birth weight reference

Appendix 7: National level estimates of neonatal deaths attributable to small for gestational age in 2012, INTERGROWTH-21^st^ standard

Appendix 8: Neonatal deaths in 2012 attributable to term-small for gestational age and preterm-small for gestational age in low and middle income countries comparing INTERGROWTH-21^st^ birth weight standard to US 1991 birth weight reference

Appendix 9: Neonatal deaths averted by reducing small for gestational age prevalence to 10% in all low and middle income countries in 2012

## Notes

We acknowledge the following individuals who contributed to the individual studies that provided data to this analysis: Ramesh Adhikari, Christian Coles, Anthony Costello, Gary Darmstadt, Sheela Devi, Subarna K Khatry, Locadiah Kuwanda, Hermann Lanou, Shabir Madhi, Tanya Marchant, Daniel Minja, Gerard I Msamanga, Robert Ntozini, David Osrin, Joanna Schellenberg, Feiko O Ter Kuile, Laeticia Celine Toe, Willy Urassa, Helen Weiss, and Nelly Zavaleta. We acknowledge the data contributed by the WHO Global Survey on Maternal and Perinatal Health Research Network. We thank Lian Folger for her extensive support in preparing the manuscript for submission. Finally, we thank the participants and patients whose data contributed to these analyses. The authors alone are responsible for the views expressed in this article and they do not necessarily represent the views, decisions, or policies of the institutions with which they are affiliated.

Contributors: ACL, NK, and JK were responsible for the study design, data collection, literature review, data analysis, and drafted the manuscript. SC, GAS, and HB provided technical inputs on the data analysis. ME, JEL, and RB provided critical input in the study design and reviewed the manuscript. MFS, AS, HER, CS, LSA, AHB, FCB, ZAB, LEC, PC, SEC, WF, RG, JH, LH, SK, PK, JL, DM, MM, AM, LCM, RN, JKN, DR, NS, DJT, JMT, CGV, SCV, DW, and BAW contributed data to the analysis and reviewed the manuscript. All authors, external and internal, had full access to all of the data (including statistical reports and tables) in the study and can take responsibility for the integrity of the data and the accuracy of the data analysis. ACL is guarantor.

Ethical approval: Datasets that were shared with the working group for secondary analysis did not contain personal identifiers and were therefore deemed exempt by the Johns Hopkins Bloomberg School of Public Health Institutional Review Board. In the original parent studies, maternal consent was obtained, and investigators were covered under individual local ethical approval.

Data sharing: The original individual cohort study data are not available for data sharing. National and regional estimates of small for gestational age are available in excel files in appendices 5 and 7.

Transparency: The lead author (ACL) affirms that the manuscript is an honest, accurate, and transparent account of the study being reported; that no important aspects of the study have been omitted; and that any discrepancies from the study as planned have been explained.
