# Supplementary material for: Estimates of burden and consequences of infants born small for gestational age in low and middle income countries with INTERGROWTH-21st standard: analysis of CHERG datasets
Source: BMJ. 2017 Aug 17;358:j3677. doi: 10.1136/bmj.j3677 (PMC5558898; doi:10.1136/bmj.j3677)
Supplement: Supplementary file 1 — Appendix 1: Studies that contributed data [file leea038389.ww1.pdf]

Appendix 1: Descriptions of studies that contributed data on SGA prevalence and/or risk ratios between SGA and neonatal / infant mortality [posted as supplied by author]

Country (cohort year)	Setting	Primary study design	Population represented	N (original cohort)	N (analyzed cohort)	NMR (per 1000 live births)	% LBW	% preterm	% SGA	% facility delivery	Method of gestational age assessment
Bangladesh (2005)[3]	Rural Sylhet, BANGLADESH	Cluster RCT of community sepsis treatment	Population-based recruitment of all pregnant women in study area	10,585	6,504	31	30	19	50	6	LMP
India (2000)[4]	Rural Tamil Nadu, INDIA	RCT of newborn Vitamin A supplementation	Population-based recruitment of all pregnant women in study area	12,936	9,506	38	33	14	62	63	LMP
Nepal (1999)[5]	Rural Sarlahi, NEPAL	Cluster RCT of multiple micronutrient supplementation	Population-based recruitment of all pregnant women in study area	4,130	3,319	42	39	22	56	6	LMP
Nepal (2003)[6]	Peri-Urban Dhanusha, NEPAL	RCT of antenatal micronutrient supplementation	Antenatal clinic-based recruitment of pregnant women in study area	1,106	1,052	25	22	7	53	53	Ultrasound
Nepal (2004)[7]	Rural Sarlahi, NEPAL	Cluster RCT of newborn skin-umbilical cord cleansing with chlorhexidine	Population based recruitment of all pregnant women in study area	23,662	21,792	32	30	18	52	10	LMP
Thailand (2001)[8]	Rural areas and Urban Bangkok, THAILAND	Prospective follow-up of birth cohort	Longitudinal birth cohort of all births in 5 districts	4,245	3,860	5	8	9	22	99	Best obstetric estimate (LMP, ultrasound or neonatal)
Burkina Faso (2004)[9]	Rural Hounde, BURKINA FASO	RCT of multiple micronutrient supplementation	Prospective, community-based cohort	1,373	1,060	21	17	16	35	77	Ultrasound at recruitment
Burkina Faso (2006)[10]	Rural Hounde, BURKINA FASO	RCT of maternal fortified food supplementation	Prospective, community-based cohort	1,316	1,067	20	16	18	29	84	Ultrasound at recruitment

Tanzania (2001)[11]	Urban Dar es Salaam, TANZANIA	RCT of multi-vitamin supplementation	Facility-based, ANC clinics	7,752	7,557	28	8	17	20	97	LMP
Tanzania (2008)[12]	Rural Korogwe, TANZANIA	Observational malaria study	Facility-based recruitment, ANC clinics, community follow-up	915	777	33	11	5	22	88	Ultrasound
Uganda (2005)[13]	Rural Kabale district, UGANDA	RCT Intermittent preventive malaria therapy and insecticide nets	Facility-based recruitment ANC clinics; only include facility births	1,561	1,477	17	7	6	10	100	Ballard external
Zimbabwe (1997)[14]	Urban Harare, ZIMBABWE	RCT of maternal -neonatal Vitamin A supplementation	Facility-based recruitment, 14 maternity clinics and hospitals	14,110	13,914	12***	14	8	33	100	Capurro
Brazil (1993)[15]	Urban Pelotas city, Rio Grande do Sul, Southern BRAZIL	Longitudinal Birth Cohort Survey	Population-based, all births in Pelotas hospitals (100% facility delivery)	5,279	4,632	7	9	11	19	100	LMP and Dubowitz
Brazil (2004)[16]	Urban Pelotas city, Rio Grande do Sul, Southern BRAZIL	Longitudinal Birth Cohort Survey	Population-based, all births in Pelotas hospitals (100% facility delivery)	4,287	3,837	10	11	16	15	100	LMP, ultrasound if available, Dubowitz

*Only contributed SGA prevalence data.

**Only contributed risk ratios between SGA and neonatal / infant mortality. Remaining datasets contributed both.

***Newborns were enrolled ≤96 hours after birth.

Abbreviations: NMR= neonatal mortality rate, LBW= low birth weight (<2500g), SGA= small-for-gestational age, LMP = Last menstrual period
