# Supplementary material for: Estimates of burden and consequences of infants born small for gestational age in low and middle income countries with INTERGROWTH-21st standard: analysis of CHERG datasets
Source: BMJ. 2017 Aug 17;358:j3677. doi: 10.1136/bmj.j3677 (PMC5558898; doi:10.1136/bmj.j3677)
Supplement: Supplementary file 2 — Appendix 2: Conversion of prevalence of small for gestational age from US 1991 reference to INTERGROWTH-21st standard [file leea038389.ww2.pdf]

Appendix 2 Conversion of Prevalence of SGA from US 1991 to Intergrowth-21st standard [posted as supplied by author]

We calculated the numbers and prevalence of Term-SGA and Preterm-SGA infants in 14 datasets using both the U.S. 1991 reference and the Intergrowth-21st standard (shown in Appendix 1). For each major geographic region, we estimated a "conversion factor" for Term-SGA and Preterm-SGA categories converting from the U.S. 1991 reference to the Intergrowth-21st standard. To calculate the conversion factor, the prevalence of term- and preterm-SGA was calculated at the individual study level using the aforementioned reference and standard. Random effects meta-analysis of proportions was performed at the regional level to calculate a pooled term- and preterm-SGA prevalence using each reference. The conversion factor was the ratio of the meta-analyzed prevalences of the Intergrowth-21st standard: U.S. 1991 reference. To calculate the proportion of Term-SGA infants (defined by Intergrowth standard) who were LBW for each major region (Africa, Asia, LAC), in each individual dataset the proportion of Term-SGA infants who were LBW was calculated and random effects meta-analysis was used to pool the data at the regional level.

Conversion factors used to calculate Intergrowth SGA prevalence (applied at country level)

	**Asia**	**Africa**	**LAC**
**Term-SGA**	0.786343612	0.668141593	0.690789474
**Preterm-SGA**	0.529411765	0.526315789	0.571428571
**Proportion of Term-SGA that is LBW**	0.506	0.459	0.370

Abbreviations:
LAC= Latin American Countries
SGA= Small-for-gestational-age
LBW= low birth weight (<2500g)
