# Supplementary material for: Estimates of burden and consequences of infants born small for gestational age in low and middle income countries with INTERGROWTH-21st standard: analysis of CHERG datasets
Source: BMJ. 2017 Aug 17;358:j3677. doi: 10.1136/bmj.j3677 (PMC5558898; doi:10.1136/bmj.j3677)
Supplement: Supplementary file 3 — Appendix 3: Pooled risk ratios for neonatal mortality in 14 CHERG datasets, with US 1991 reference and INTERGROWTH-21st standard [file leea038389.ww3.pdf]

Appendix 3 Pooled risk ratios for neonatal mortality in 14 CHERG datasets, using the US 1991 reference and Intergrowth standard [posted as supplied by author]

	US 1991	Intergrowth
Asia		
TSGA-not LBW	1.18 (0.72, 1.92)	1.08 (0.67, 1.73)
TSGA-LBW	2.88 (2.12, 3.92)	2.57 (1.96, 3.38)
PSGA	10.29 (7.26, 14.60)	13.81 (10.26, 18.61)
PAGA	3.16 (2.19, 4.54)	2.97 (2.38, 3.69)
Asia		
TSGA-not LBW	1.38 (1.01, 1.88)	2.62 (1.43, 4.80)
TSGA-LBW	3.83 (2.78, 5.28)	4.26 (3.09, 5.87)
PSGA	9.53 (6.82, 13.30)	10.51 (6.81, 16.22)
PAGA	5.55 (2.87, 10.71)	6.12 (3.53, 10.63)
Americas		
TSGA-not LBW	2.38 (0.61, 9.27)	3.63 (0.94, 14.05)
TSGA-LBW	8.59 (2.55, 28.95)	10.34 (3.31, 32.32)
PSGA	35.65 (14.91, 85.28)	75.28 (30.48, 185.96)
PAGA	17.81 (9.51, 33.35)	18.13 (9.62, 34.16)

Abbreviations:
TSGA= Term and Small-for-gestational-age; PSGA= Preterm and Small-for-gestational-age
TAGA= Term and appropriate-for-gestational-age; PAGA= Preterm and appropriate-for-gestational-age
LBW= low birth weight (<2500g)
