# Supplementary material for: Estimates of burden and consequences of infants born small for gestational age in low and middle income countries with INTERGROWTH-21st standard: analysis of CHERG datasets
Source: BMJ. 2017 Aug 17;358:j3677. doi: 10.1136/bmj.j3677 (PMC5558898; doi:10.1136/bmj.j3677)
Supplement: Supplementary file 4 — Appendix 4: Methods of uncertainty estimation [file leea038389.ww4.pdf]

Appendix 4 Methods of Uncertainty Estimation [posted as supplied by author]

To estimate uncertainty ranges (URs), we used a bootstrap approach to randomly draw 1000 point estimates of: 1) the prevalences of Term-SGA-LBW, Term-SGA-not-LBW, and Preterm-SGA from the original 1991 U.S. population reference[1], 2) conversion factors from the U.S. 1991 population reference to Intergrowth 21st standard (by major UN region), and 3) the risk ratio of neonatal mortality for Term-SGA-LBW, Term-SGA-not-LBW, Preterm-AGA, and Preterm-SGA (with reference group Term-AGA) for the major UN region of the country. The sources of bootstrapped estimates for the original SGA prevalences (1991 U.S. population) for all categories are described in detail in a prior publication.[1]

To obtain the bootstrapped estimates of the SGA prevalence using the Intergrowth 21st standard, we first obtained 1000 samples of the conversion factors going from the U.S. 1991 population to Intergrowth, by randomly choosing a point estimate (proportion) from the normal distribution of the conversion factor. These 1000 samples of conversion factors were multiplied by the 1000 bootstrapped samples of SGA prevalence (U.S. 1991 reference) from the original modeling dataset.[1]

For risk ratios, we randomly chose a point estimate from the normal distribution with mean and standard deviation per the pooled meta-estimate for each respective risk factor-outcome association, with each country drawing from the major UN pooled regional risk and distribution. We then used each set of randomly sampled prevalences and risk ratios to calculate 1000 population attributable fractions (PAFs). If any randomly generated PAF was <0, the PAF for that sample was set to 0.

Uncertainty ranges were calculated at the national and regional levels for SGA births and attributable neonatal deaths. The uncertainty range for a country was defined as the 2.5^{th} and 97.5^{th} percentile of the 1000 simulated point estimates. To generate regional uncertainty, national estimates were summed at the regional level for the 1000 bootstrapped samples, and the 2.5^{th} and 97.5^{th} percentile estimates were chosen. Uncertainty in the overall mortality estimates[2] was not accounted for.
