# Supplementary material for: Estimates of burden and consequences of infants born small for gestational age in low and middle income countries with INTERGROWTH-21st standard: analysis of CHERG datasets
Source: BMJ. 2017 Aug 17;358:j3677. doi: 10.1136/bmj.j3677 (PMC5558898; doi:10.1136/bmj.j3677)
Supplement: Supplementary file 5 — Appendix 5: National level estimates of small for gestational age prevalence in 2012, INTERGROWTH-21st standard [file leea038389.ww5.pdf]

Appendix 5:

National-level Estimates of Small-for-Gestational-Age (SGA) Birth Prevalence for 138 LMIC Countries for 2012, using the INTERGROWTH 21st standard [posted as supplied by author]

Version June 27, 2017

							Birth Prevalence in 2012									Number of Births in 2012								
isocode	country	WHO region	GBD region	UN-MDG Region	Live births 2012* (N)	NMR 2012**	Preterm Birth Prevalence (%) 2012***	SGA Prevalence (%)	Uncertainty Range (UR)	Term-SGA-not-LBW Prevalence (%)	UR	Term-SGA-LBW Prevalence (%)	UR	Preterm-SGA Prevalence (%)	UR	Preterm birth	Total SGA births (N)	Uncertainty Range (UR)	Term-SGA-not LBW (N)	UR	Term-SGA-LBW (N)	UR	Preterm-SGA (N)	UR
AFG	Afghanistan	EMRO	Asia South	Southern Asia	1000000	36	11.5	30.8	(21.8 to 46.3)	14.5	(32.3 to 42.6)	14.9	(32.3 to 42.6)	1.4	(1 to 5.3)	118900	318100	(218000 to 462700)	150200	(98500 to 221300)	153800	(101000 to 229000)	14100	(4800 to 34900)
DZA	Algeria	AFRO	North Africa/Middle East	Northern Africa	996086	11.6	7.4	6.9	(3.9 to 12.6)	3.4	(6.7 to 12.8)	2.9	(6.7 to 12.8)	0.6	(0.7 to 2.3)	73700	68900	(38900 to 125500)	33900	(16800 to 64300)	28800	(14300 to 55800)	6200	(2500 to 15600)
AGO	Angola	AFRO	Sub-Saharan Africa Central	Sub-Saharan Africa	906908	45.4	12.5	16.8	(9.9 to 28.5)	8.5	(20 to 27.2)	7.2	(20 to 27.2)	1.1	(0.8 to 4.1)	113400	152500	(89400 to 258000)	77300	(42100 to 139500)	65600	(34400 to 117300)	9500	(3100 to 24800)
ATG	Antigua and Barbuda	AMRO	Caribbean	Latin America and the Caribbean	1471	5.5	5.8	6.1	(4.7 to 13.6)	3.3	(5 to 11.3)	2.0	(5 to 11.3)	0.7	(0.8 to 2.5)	100	89	(70 to 200)	49	(36 to 100)	29	(22 to 70)	11	(6 to 28)
ARG	Argentina	AMRO	Latin America Southern	Latin America and the Caribbean	705590	7.5	8.0	7.6	(6.3 to 16.6)	4.2	(6.6 to 13.4)	2.5	(6.6 to 13.4)	1.0	(1.5 to 1.9)	56400	53500	(44700 to 117300)	29500	(22500 to 67800)	17300	(13400 to 40100)	6700	(4900 to 12600)
ARM	Armenia	EURO	Asia Central	Caucasus and Central Asia	41695	10.1	11.0	12.3	(7.7 to 19.6)	5.4	(9.4 to 20.1)	5.5	(9.4 to 20.1)	1.3	(1 to 5.3)	4600	5100	(3200 to 8200)	2300	(1300 to 3800)	2300	(1300 to 3900)	500	(200 to 1400)
AZE	Azerbaijan	EURO	Asia Central	Caucasus and Central Asia	170955	15	8.5	12.3	(7.7 to 19.7)	5.6	(9.8 to 20.6)	5.7	(9.8 to 20.6)	1.0	(1 to 4.1)	14500	21000	(13200 to 33700)	9500	(5500 to 15900)	9700	(5700 to 16300)	1700	(700 to 4200)
BHS	Bahamas	AMRO	Caribbean	Latin America and the Caribbean	6082	7.7	9.5	11.3	(9.6 to 24)	6.4	(10.6 to 19.3)	3.8	(10.6 to 19.3)	1.1	(1.3 to 3.7)	600	700	(600 to 1500)	400	(300 to 900)	200	(200 to 500)	68	(45 to 200)
BHR	Bahrain	EMRO	North Africa/Middle East	Western Asia	20474	3.6	14.0	14.6	(9.3 to 22.8)	6.4	(11.6 to 22.8)	6.5	(11.6 to 22.8)	1.6	(2 to 4.2)	2900	3000	(1900 to 4700)	1300	(800 to 2100)	1300	(800 to 2200)	300	(200 to 600)
BGD	Bangladesh	SEARO	Asia South	Southern Asia	3100000	24.4	14.0	30.3	(20.8 to 43.9)	14.2	(32.6 to 42.1)	14.5	(32.6 to 42.1)	1.6	(2.2 to 4.3)	435500	943900	(644100 to 1361600)	441000	(288200 to 648300)	451700	(295200 to 668000)	51300	(25500 to 107000)
BRB	Barbados	AMRO	Caribbean	Latin America and the Caribbean	3644	9.5	8.9	11.7	(9.9 to 24.5)	6.6	(11.1 to 19.7)	3.9	(11.1 to 19.7)	1.2	(1.2 to 3.3)	300	400	(400 to 900)	200	(200 to 500)	100	(100 to 300)	43	(25 to 100)
BLZ	Belize	AMRO	Caribbean	Latin America and the Caribbean	7750	8.6	10.4	13.6	(11.7 to 28.9)	7.8	(13.1 to 23)	4.6	(13.1 to 23)	1.2	(1.5 to 4)	800	1100	(900 to 2200)	600	(500 to 1300)	400	(300 to 800)	96	(62 to 300)
BEN	Benin	AFRO	Sub-Saharan Africa West	Sub-Saharan Africa	357338	27.8	10.6	17.0	(9.9 to 28.9)	8.7	(20 to 27.6)	7.4	(20 to 27.6)	0.9	(0.9 to 3.6)	37900	60700	(35400 to 103100)	31100	(16600 to 56500)	26400	(13800 to 47300)	3200	(1300 to 8000)
BTN	Bhutan	SEARO	Asia South	Southern Asia	14850	21.2	10.2	16.5	(10.6 to 25.2)	7.6	(14.5 to 25.6)	7.7	(14.5 to 25.6)	1.2	(0.9 to 5)	1500	2400	(1600 to 3700)	1100	(700 to 1800)	1100	(700 to 1800)	200	(65 to 500)
BOL	Bolivia	AMRO	Latin America Andean	Latin America and the Caribbean	263587	18.9	8.1	6.6	(5 to 14)	3.5	(5.4 to 11.6)	2.1	(5.4 to 11.6)	1.0	(0.4 to 1.6)	23700	17300	(13100 to 36900)	9300	(7200 to 22200)	5500	(4200 to 13100)	2600	(700 to 3400)
BWA	Botswana	AFRO	Sub-Saharan Africa Southern	Sub-Saharan Africa	48531	28.5	15.1	13.3	(7.6 to 22.6)	6.5	(12.9 to 22.8)	5.5	(12.9 to 22.8)	1.3	(0.9 to 4)	7300	6400	(3700 to 11000)	3200	(1600 to 5700)	2700	(1300 to 4800)	600	(200 to 1500)
BRA	Brazil	AMRO	Latin America Tropical	Latin America and the Caribbean	2900000	9.2	9.2	9.0	(7.5 to 19.5)	5.0	(8.2 to 15.5)	2.9	(8.2 to 15.5)	1.1	(1.2 to 3.1)	266500	261700	(218600 to 565800)	144700	(113500 to 323900)	85000	(67400 to 192500)	31900	(19200 to 72200)
BRN	Brunei Darussalam	WPRO	Asia Pacific High Income	South-eastern Asia	6209	4.4	12.1	15.6	(10.1 to 24.5)	7.0	(13.1 to 24.6)	7.2	(13.1 to 24.6)	1.4	(1.2 to 6.1)	800	1000	(600 to 1500)	400	(300 to 700)	400	(300 to 700)	87	(29 to 200)
BFA	Burkina Faso	AFRO	Sub-Saharan Africa West	Sub-Saharan Africa	655851	27.5	10.9	13.7	(8.1 to 22.9)	6.9	(15.9 to 22.3)	5.8	(15.9 to 22.3)	0.9	(1 to 2.6)	71500	89600	(53300 to 150300)	45200	(24500 to 81300)	38300	(19800 to 68200)	6000	(2500 to 13000)
BDI	Burundi	AFRO	Sub-Saharan Africa East	Sub-Saharan Africa	428114	35.5	11.4	15.5	(8.8 to 26.4)	7.9	(18.4 to 25.2)	6.7	(18.4 to 25.2)	1.0	(0.7 to 3.9)	48800	66400	(37700 to 113200)	33700	(18400 to 61200)	28600	(14900 to 51200)	4100	(1300 to 11400)
KHM	Cambodia	WPRO	Asia Southeast	South-eastern Asia	362078	18.4	10.5	14.1	(9.3 to 22.1)	6.4	(11.6 to 22.5)	6.5	(11.6 to 22.5)	1.2	(1 to 5.2)	38000	51200	(33600 to 80000)	23000	(13400 to 37600)	23600	(14200 to 38800)	4500	(1500 to 12000)
CMR	Cameroon	AFRO	Sub-Saharan Africa West	Sub-Saharan Africa	787199	27.9	12.6	13.8	(8 to 23.1)	6.9	(15.9 to 22.4)	5.9	(15.9 to 22.4)	1.1	(1 to 3.7)	99200	109000	(63300 to 182100)	54500	(29400 to 98100)	46200	(23900 to 82200)	8400	(3000 to 18800)
CPV	Cape Verde	AFRO	Sub-Saharan Africa West	Sub-Saharan Africa	10120	10	11.2	7.0	(4 to 12.4)	3.2	(6.2 to 12.5)	2.8	(6.2 to 12.5)	1.0	(0.7 to 3)	1100	700	(400 to 1300)	300	(200 to 600)	300	(100 to 500)	100	(33 to 200)
CAF	Central African Republic	AFRO	Sub-Saharan Africa Central	Sub-Saharan Africa	150396	40.9	12.6	17.8	(10.4 to 30.5)	9.0	(21.1 to 28.7)	7.7	(21.1 to 28.7)	1.1	(0.8 to 4.6)	18900	26700	(15600 to 45900)	13600	(7400 to 24200)	11500	(6000 to 20700)	1600	(500 to 4400)
TCD	Chad	AFRO	Sub-Saharan Africa West	Sub-Saharan Africa	554832	39.7	13.1	24.3	(14 to 40.8)	12.5	(29.5 to 39.8)	10.6	(29.5 to 39.8)	1.1	(0.8 to 4.5)	72700	134700	(77500 to 226400)	69600	(37000 to 122500)	59000	(30900 to 108800)	6100	(2000 to 16300)
CHL	Chile	AMRO	Latin America Southern	Latin America and the Caribbean	246377	5.3	7.1	6.4	(5.3 to 14.5)	3.5	(5.3 to 11.7)	2.1	(5.3 to 11.7)	0.8	(1.3 to 1.7)	17500	15900	(13000 to 35700)	8700	(6400 to 20300)	5100	(3800 to 12100)	2100	(1500 to 3900)
CHN	China	WPRO	Asia East	Eastern Asia	19000000	8.5	7.1	4.7	(2.6 to 8.4)	1.9	(2.3 to 9.2)	1.9	(2.3 to 9.2)	0.8	(1.1 to 2)	1315000	865600	(485400 to 1600100)	351400	(152100 to 731400)	359900	(160000 to 736100)	154400	(77300 to 299400)
COL	Colombia	AMRO	Latin America Central	Latin America and the Caribbean	914535	11.2	8.8	9.6	(8.2 to 20.6)	5.4	(9 to 16.4)	3.2	(9 to 16.4)	1.1	(1.3 to 2.2)	80500	87800	(74800 to 188500)	49300	(39400 to 109600)	28900	(23300 to 64900)	9600	(6400 to 18300)
COM	Comoros	AFRO	Sub-Saharan Africa East	Sub-Saharan Africa	24930	31	16.7	24.8	(14 to 41.3)	12.6	(28.8 to 40.5)	10.7	(28.8 to 40.5)	1.4	(1.1 to 5)	4200	6200	(3500 to 10300)	3100	(1600 to 5600)	2700	(1400 to 4900)	400	(100 to 800)
COG	Congo	AFRO	Sub-Saharan Africa Central	Sub-Saharan Africa	162254	31.7	16.7	16.1	(9.7 to 26.3)	7.9	(18.2 to 25.4)	6.7	(18.2 to 25.4)	1.4	(1.1 to 4.2)	27100	26100	(15700 to 42700)	12900	(6900 to 23400)	10900	(5700 to 19500)	2300	(800 to 5200)
CRI	Costa Rica	AMRO	Latin America Central	Latin America and the Caribbean	72676	6.6	13.6	7.5	(6 to 16.2)	3.7	(5.6 to 12.1)	2.2	(5.6 to 12.1)	1.6	(1.2 to 5.1)	9900	5400	(4400 to 11800)	2700	(2000 to 6200)	1600	(1200 to 3700)	1200	(500 to 2900)
CIV	Cote d'Ivoire	AFRO	Sub-Saharan Africa West	Sub-Saharan Africa	714453	39.9	14.0	20.4	(11.8 to 34)	10.4	(24.6 to 32.7)	8.8	(24.6 to 32.7)	1.2	(0.9 to 4.7)	100000	145700	(84500 to 243100)	74300	(40100 to 132300)	63000	(33200 to 112900)	8500	(2500 to 23700)
CUB	Cuba	AMRO	Caribbean	Latin America and the Caribbean	101941	2.6	6.4	5.6	(4.5 to 12.9)	3.1	(4.5 to 10.5)	1.8	(4.5 to 10.5)	0.8	(1.1 to 1.7)	6500	5800	(4600 to 13100)	3100	(2300 to 7500)	1800	(1400 to 4500)	800	(500 to 1500)
PRK	Democratic People's Republic of Korea	SEARO	Asia East	Eastern Asia	346634	15.6	10.7	12.0	(7.4 to 19.5)	5.3	(9.2 to 19.9)	5.4	(9.2 to 19.9)	1.3	(1 to 5)	37100	41600	(25800 to 67500)	18400	(10500 to 30900)	18800	(10800 to 31800)	4400	(1500 to 11900)
COD	Democratic Republic of the Congo	AFRO	Sub-Saharan Africa Central	Sub-Saharan Africa	2700000	43.5	11.9	14.4	(8.7 to 24.9)	7.2	(16.3 to 23.6)	6.1	(16.3 to 23.6)	1.0	(0.8 to 4)	323100	390200	(234000 to 672700)	196300	(108600 to 360000)	166600	(86700 to 301500)	27300	(8600 to 75200)
DJI	Djibouti	EMRO	Sub-Saharan Africa East	Sub-Saharan Africa	23582	31.4	11.9	21.3	(12.3 to 35.5)	11.0	(25.2 to 34.7)	9.3	(25.2 to 34.7)	1.0	(0.7 to 4)	2800	5000	(2900 to 8400)	2600	(1400 to 4500)	2200	(1200 to 4000)	200	(69 to 600)
DMA	Dominica	AMRO	Caribbean	Latin America and the Caribbean	1072	9	11.9	9.8	(8.6 to 22.3)	5.6	(9.2 to 17.1)	3.3	(9.2 to 17.1)	1.0	(1.4 to 5.6)	100	100	(93 to 200)	60	(48 to 100)	35	(28 to 78)	11	(9 to 46)
DOM	Dominican Republic	AMRO	Caribbean	Latin America and the Caribbean	214493	15	10.8	9.0	(7.5 to 19.4)	4.8	(8 to 14.8)	2.8	(8 to 14.8)	1.3	(1.2 to 4.7)	23200	19200	(16100 to 41700)	10400	(8200 to 23300)	6100	(4800 to 13800)	2800	(1500 to 7500)
ECU	Ecuador	AMRO	Latin America Andean	Latin America and the Caribbean	324415	10.3	5.1	6.6	(5.3 to 14.7)	3.8	(5.8 to 12.2)	2.2	(5.8 to 12.2)	0.6	(0.5 to 2.6)	16500	21300	(17300 to 47800)	12200	(9300 to 28300)	7200	(5500 to 16700)	2000	(1000 to 6600)
EGY	Egypt	EMRO	North Africa/Middle East	Northern Africa	1900000	11.8	7.3	6.5	(3.6 to 12.1)	3.2	(5.8 to 12.2)	2.7	(5.8 to 12.2)	0.6	(0.5 to 2)	140700	124400	(68400 to 229400)	60900	(29800 to 118000)	51700	(25000 to 102100)	11900	(4100 to 27500)
SLV	El Salvador	AMRO	Latin America Central	Latin America and the Caribbean	135842	6.3	12.8	10.3	(8.3 to 22.2)	5.5	(9 to 16.9)	3.2	(9 to 16.9)	1.5	(1.1 to 6.3)	17400	13900	(11300 to 30200)	7500	(5900 to 16600)	4400	(3500 to 9900)	2100	(900 to 6800)
GNQ	Equatorial Guinea	AFRO	Sub-Saharan Africa Central	Sub-Saharan Africa	25496	34.1	16.5	16.7	(9.7 to 27.8)	8.3	(19.3 to 26.2)	7.0	(19.3 to 26.2)	1.4	(1.1 to 5.1)	4200	4200	(2500 to 7100)	2100	(1100 to 3800)	1800	(900 to 3200)	400	(100 to 1000)
ERI	Eritrea	AFRO	Sub-Saharan Africa East	Sub-Saharan Africa	221678	18.2	12.2	15.1	(8.7 to 25.9)	7.6	(16.4 to 25.3)	6.5	(16.4 to 25.3)	1.0	(0.9 to 4)	27000	33500	(19200 to 57300)	16900	(8800 to 30200)	14300	(7300 to 25600)	2300	(800 to 6100)
ETH	Ethiopia	AFRO	Sub-Saharan Africa East	Sub-Saharan Africa	3000000	29	10.1	21.2	(12.2 to 35.9)	11.0	(25.4 to 35.1)	9.3	(25.4 to 35.1)	0.9	(0.9 to 2.3)	305900	642200	(365900 to 1076900)	333400	(174700 to 582400)	282900	(148000 to 511300)	25800	(10300 to 51400)
FJI	Fiji	WPRO	Oceana	Oceania	18397	9.9	9.9	13.6	(8.7 to 21.7)	6.2	(11.1 to 22)	6.3	(11.1 to 22)	1.1	(0.9 to 4.3)	1800	2500	(1600 to 4000)	1100	(700 to 1900)	1200	(700 to 1900)	200	(79 to 600)
GAB	Gabon	AFRO	Sub-Saharan Africa Central	Sub-Saharan Africa	52030	24.9	16.3	16.0	(9.5 to 26.9)	7.9	(17.5 to 25.8)	6.7	(17.5 to 25.8)	1.4	(1.1 to 4.9)	8500	8300	(5000 to 14000)	4100	(2200 to 7400)	3500	(1800 to 6300)	700	(200 to 1800)
GMB	Gambia	AFRO	Sub-Saharan Africa West	Sub-Saharan Africa	74548	28	14.0	21.6	(12.7 to 35.8)	11.1	(25.5 to 34.9)	9.4	(25.5 to 34.9)	1.2	(0.9 to 4.9)	10400	16100	(9400 to 26700)	8200	(4300 to 14500)	7000	(3700 to 12500)	900	(300 to 2300)
GEO	Georgia	EURO	Asia Central	Caucasus and Central Asia	61453	14.7	8.8	11.6	(7.2 to 18.7)	5.2	(9 to 19.7)	5.4	(9 to 19.7)	1.0	(1.1 to 2.9)	5400	7200	(4500 to 11500)	3200	(1800 to 5400)	3300	(1900 to 5600)	600	(300 to 1300)
GHA	Ghana	AFRO	Sub-Saharan Africa West	Sub-Saharan Africa	782917	28.4	14.5	15.9	(9.4 to 26.5)	7.9	(18.1 to 25.4)	6.7	(18.1 to 25.4)	1.2	(0.9 to 4.9)	113500	124200	(73800 to 207100)	62000	(33000 to 112800)	52600	(27400 to 94000)	9600	(3100 to 26100)
GRD	Grenada	AMRO	Caribbean	Latin America and the Caribbean	2135	6.7	10.3	8.1	(6.8 to 17.6)	4.3	(6.9 to 13.8)	2.5	(6.9 to 13.8)	1.2	(1.2 to 4.6)	200	200	(100 to 400)	93	(71 to 200)	54	(42 to 100)	25	(15 to 77)
GTM	Guatemala	AMRO	Latin America Central	Latin America and the Caribbean	465899	15.3	7.7	9.2	(7.7 to 19.7)	5.2	(8.7 to 15.9)	3.1	(8.7 to 15.9)	0.9	(1 to 2.7)	35900	42800	(35800 to 92000)	24300	(19500 to 54200)	14200	(11400 to 32000)	4300	(2700 to 10100)
GIN	Guinea	AFRO	Sub-Saharan Africa West	Sub-Saharan Africa	413720	34.4	13.9	16.2	(9.8 to 27.4)	8.1	(19.1 to 25.7)	6.9	(19.1 to 25.7)	1.2	(0.9 to 4.8)	57500	66900	(40300 to 113400)	33600	(18200 to 60900)	28500	(14900 to 50800)	4900	(1600 to 13100)
GNB	Guinea-Bissau	AFRO	Sub-Saharan Africa West	Sub-Saharan Africa	60230	45.7	11.2	25.9	(14.9 to 43.8)	13.5	(31.8 to 42.9)	11.4	(31.8 to 42.9)	0.9	(0.7 to 3.7)	6700	15600	(9000 to 26400)	8100	(4300 to 14400)	6900	(3600 to 12600)	600	(200 to 1500)
GUY	Guyana	AMRO	Caribbean	Latin America and the Caribbean	15990	19.4	13.2	12.9	(11.2 to 27)	7.1	(12.4 to 20.5)	4.2	(12.4 to 20.5)	1.6	(1.6 to 6)	2100	2100	(1800 to 4300)	1100	(900 to 2500)	700	(600 to 1500)	300	(100 to 700)
HTI	Haiti	AMRO	Caribbean	Latin America and the Caribbean	265532	25.4	14.1	23.7	(20.3 to 49.2)	13.9	(25.1 to 38.5)	8.1	(25.1 to 38.5)	1.7	(1.7 to 6.3)	37400	62900	(53900 to 130700)	36800	(30600 to 78000)	21600	(18100 to 46400)	4500	(2500 to 13600)
HND	Honduras	AMRO	Latin America Central	Latin America and the Caribbean	206618	11.5	12.2	10.9	(9.4 to 23.6)	6.0	(10 to 18)	3.5	(10 to 18)	1.4	(1.4 to 5.7)	25200	22600	(19300 to 48800)	12300	(9900 to 27200)	7200	(5800 to 16100)	3000	(1700 to 8900)
IND	India	SEARO	Asia South	Southern Asia	25000000	30.9	13.0	36.1	(25 to 52.8)	17.1	(40 to 50)	17.5	(40 to 50)	1.5	(2 to 3.8)	3277200	9111700	(6244800 to 1320000	4311000	(2796500 to 634860	4415700	(2892100 to 6480000)	385000	(187900 to 771600)
IDN	Indonesia	SEARO	Asia Southeast	South-eastern Asia	4800000	15	15.5	17.9	(12 to 27.3)	7.9	(15.4 to 26.3)	8.1	(15.4 to 26.3)	1.8	(1.9 to 4.8)	748500	862600	(575300 to 1308800)	382600	(233600 to 593800)	391900	(246200 to 617900)	88100	(40100 to 183000)
IRN	Iran (Islamic Republic of)	EMRO	North Africa/Middle East	Southern Asia	1500000	10.8	12.9	12.6	(8.1 to 19.8)	5.4	(9.3 to 19.7)	5.6	(9.3 to 19.7)	1.5	(1.8 to 4)	192800	187700	(121700 to 297200)	81400	(47000 to 136200)	83400	(48600 to 140100)	22800	(11100 to 45800)
IRQ	Iraq	EMRO	North Africa/Middle East	Western Asia	1000000	19	6.5	10.2	(6.3 to 17.1)	4.7	(7.9 to 18.6)	4.8	(7.9 to 18.6)	0.8	(0.7 to 3.3)	66200	104100	(62600 to 171100)	47600	(26000 to 81600)	48700	(27100 to 84500)	7900	(2800 to 21300)
JAM	Jamaica	AMRO	Caribbean	Latin America and the Caribbean	53923	10.6	10.2	12.3	(10.5 to 26.1)	7.0	(11.7 to 20.4)	4.1	(11.7 to 20.4)	1.2	(1.4 to 4.9)	5500	6600	(5700 to 14100)	3700	(3100 to 8300)	2200	(1800 to 4900)	700	(400 to 2000)
JOR	Jordan	EMRO	North Africa/Middle East	Western Asia	200412	11.5	14.4	14.0	(9 to 22.1)	6.1	(11 to 21.8)	6.2	(11 to 21.8)	1.7	(1.1 to 5)	28900	28100	(18100 to 44300)	12200	(7100 to 20100)	12500	(7500 to 20800)	3400	(1000 to 7600)
KAZ	Kazakhstan	EURO	Asia Central	Caucasus and Central Asia	344626	10.1	8.8	12.5	(7.8 to 19.9)	5.7	(10 to 20.8)	5.8	(10 to 20.8)	1.0	(1.2 to 2.6)	30300	43100	(26900 to 68700)	19500	(11300 to 32600)	20000	(11700 to 33500)	3600	(1700 to 6900)
KEN	Kenya	AFRO	Sub-Saharan Africa East	Sub-Saharan Africa	1500000	26.8	12.3	10.1	(5.8 to 17.3)	4.9	(10.8 to 17.3)	4.1	(10.8 to 17.3)	1.0	(1.1 to 2.9)	183600	150300	(87700 to 260100)	72900	(37900 to 132400)	61900	(32300 to 114300)	15500	(6300 to 32600)
KIR	Kiribati	WPRO	Oceana	Oceania	2377	21.6	9.6	14.2	(9 to 22.2)	6.4	(11.7 to 22.7)	6.6	(11.7 to 22.7)	1.2	(0.9 to 4.5)	200	300	(200 to 500)	200	(89 to 200)	200	(95 to 300)	28	(9 to 72)
KWT	Kuwait	EMRO	North Africa/Middle East	Western Asia	68280	5.5	10.6	11.8	(7.2 to 18.9)	5.2	(8.9 to 19.5)	5.3	(8.9 to 19.5)	1.2	(0.9 to 4.9)	7200	8000	(4900 to 12900)	3500	(2000 to 6000)	3600	(2100 to 6200)	900	(300 to 2300)
KGZ	Kyrgyzstan	EURO	Asia Central	Caucasus and Central Asia	150386	14.2	10.4	11.1	(6.8 to 18)	4.9	(8.3 to 18.8)	5.0	(8.3 to 18.8)	1.2	(1 to 4.8)	15600	16700	(10200 to 27000)	7300	(4100 to 12600)	7500	(4300 to 12900)	1800	(700 to 4700)
LAO	Lao People's Democratic Republic	WPRO	Asia Southeast	South-eastern Asia	193528	27.2	10.8	17.3	(11.4 to 26.6)	7.9	(15.5 to 26.5)	8.1	(15.5 to 26.5)	1.3	(1.1 to 5.5)	20900	33500	(22100 to 51500)	15300	(9400 to 23900)	15700	(9900 to 24600)	2500	(900 to 6600)
LBN	Lebanon	EMRO	North Africa/Middle East	Western Asia	59684	5.4	7.9	9.5	(5.7 to 15.9)	4.2	(6.8 to 16.8)	4.3	(6.8 to 16.8)	0.9	(1.1 to 2.3)	4700	5700	(3400 to 9500)	2500	(1400 to 4400)	2600	(1400 to 4500)	600	(300 to 1100)
LSO	Lesotho	AFRO	Sub-Saharan Africa Southern	Sub-Saharan Africa	57877	45.3	11.9	14.2	(8.4 to 24.2)	7.1	(16.5 to 23)	6.1	(16.5 to 23)	1.0	(0.7 to 4)	6900	8200	(4800 to 14000)	4100	(2300 to 7600)	3500	(1800 to 6200)	600	(200 to 1600)
LBR	Liberia	AFRO	Sub-Saharan Africa West	Sub-Saharan Africa	146907	26.6	13.9	15.9	(9.2 to 26.5)	7.9	(18 to 25.6)	6.7	(18 to 25.6)	1.2	(0.9 to 4.8)	20400	23300	(13500 to 39000)	11700	(6100 to 21300)	9900	(5100 to 17700)	1700	(600 to 4700)
LBY	Libyan Arab Jamahiriya	EMRO	North Africa/Middle East	Northern Africa	128708	9.4	8.3	4.8	(2.6 to 9.2)	2.2	(3.9 to 9.3)	1.9	(3.9 to 9.3)	0.7	(0.5 to 2.8)	10700	6200	(3400 to 11800)	2900	(1400 to 5800)	2400	(1100 to 5000)	900	(300 to 2400)
MDG	Madagascar	AFRO	Sub-Saharan Africa East	Sub-Saharan Africa	768940	22	14.2	16.7	(9.7 to 28.4)	8.4	(18.4 to 27.3)	7.1	(18.4 to 27.3)	1.2	(0.9 to 4.7)	109200	128500	(74400 to 218100)	64500	(33500 to 115900)	54700	(28000 to 98400)	9200	(2900 to 23700)
MWI	Malawi	AFRO	Sub-Saharan Africa East	Sub-Saharan Africa	617027	24.2	18.1	15.1	(8.9 to 25)	7.3	(16.5 to 24)	6.2	(16.5 to 24)	1.5	(1.7 to 4.3)	111700	93200	(55200 to 154200)	45300	(24100 to 82000)	38500	(19800 to 68700)	9400	(4100 to 20100)
MYS	Malaysia	WPRO	Asia Southeast	South-eastern Asia	503080	4.5	12.3	15.6	(9.7 to 24.5)	7.0	(13 to 24.5)	7.2	(13 to 24.5)	1.5	(1.2 to 5.7)	61900	78600	(49000 to 123200)	35200	(20600 to 56400)	36100	(21700 to 58500)	7300	(2400 to 18500)
MDV	Maldives	SEARO	Asia Southeast	Southern Asia	7990	6.3	7.9	14.3	(8.9 to 22.7)	6.6	(12.3 to 23.3)	6.8	(12.3 to 23.3)	0.9	(1 to 2.4)	600	1100	(700 to 1800)	500	(300 to 900)	500	(300 to 900)	71	(34 to 200)
MLI	Mali	AFRO	Sub-Saharan Africa West	Sub-Saharan Africa	670362	41.5	11.6	23.3	(13.5 to 40)	12.1	(28.5 to 38.5)	10.2	(28.5 to 38.5)	1.0	(0.8 to 4.1)	77800	155900	(90600 to 268100)	80800	(43700 to 142700)	68500	(35600 to 124900)	6600	(2100 to 19600)
MHL	Marshall Islands	WPRO	Oceana	Oceania	1480	15.8	11.5	21.0	(14 to 31.6)	9.8	(20.2 to 31.4)	10.1	(20.2 to 31.4)	1.1	(1 to 5.4)	200	300	(200 to 500)	100	(91 to 200)	100	(93 to 200)	16	(7 to 50)
MRT	Mauritania	AFRO	Sub-Saharan Africa West	Sub-Saharan Africa	127034	33.6	15.4	31.1	(17.8 to 52.1)	16.1	(37.2 to 52.2)	13.7	(37.2 to 52.2)	1.3	(1 to 4.8)	19600	39400	(22600 to 66200)	20400	(10600 to 36900)	17300	(8900 to 31700)	1655	(500 to 4100)
MUS	Mauritius	AFRO	Asia Southeast	Sub-Saharan Africa	13284	9.3	12.6	13.4	(7.6 to 23.5)	6.7	(12.9 to 23.6)	5.6	(12.9 to 23.6)	1.1	(0.8 to 4.2)	1700	1800	(1000 to 3100)	900	(400 to 1600)	800	(400 to 1400)	100	(44 to 400)
MEX	Mexico	AMRO	Latin America Central	Latin America and the Caribbean	2300000	7.2	7.3	6.6	(5.4 to 14.6)	3.6	(5.6 to 12.1)	2.1	(5.6 to 12.1)	0.9	(1.3 to 1.8)	166200	151100	(125000 to 336700)	82600	(62500 to 192900)	48500	(36400 to 114100)	19900	(14500 to 37100)
FSM	Micronesia (Federated States of)	WPRO	Oceana	Oceania	2414	16.2	10.5	17.6	(11.5 to 27)	8.0	(15.8 to 26.7)	8.2	(15.8 to 26.7)	1.3	(1 to 4.9)	300	400	(300 to 700)	200	(100 to 300)	200	(100 to 300)	32	(10 to 76)
MNG	Mongolia	WPRO	Asia Central	Eastern Asia	66641	9.7	13.5	7.7	(4.7 to 13.1)	3.0	(4.4 to 13.2)	3.1	(4.4 to 13.2)	1.6	(1.4 to 4.8)	9000	5100	(3100 to 8700)	2000	(1000 to 3800)	2100	(1000 to 3800)	1100	(400 to 2300)
MAR	Morocco	EMRO	North Africa/Middle East	Northern Africa	746689	17.8	6.7	4.9	(2.7 to 9.2)	2.4	(4.2 to 9.7)	2.0	(4.2 to 9.7)	0.6	(0.4 to 2.5)	50000	36900	(19900 to 68900)	17700	(8500 to 34800)	15000	(7100 to 29800)	4200	(1300 to 11500)
MOZ	Mozambique	AFRO	Sub-Saharan Africa East	Sub-Saharan Africa	936353	30.2	16.4	18.2	(10.9 to 30.4)	9.1	(21.2 to 28.6)	7.7	(21.2 to 28.6)	1.4	(1.1 to 4.2)	153600	170800	(102300 to 285000)	85400	(45700 to 152600)	72400	(37900 to 129400)	13000	(4600 to 28700)
MMR	Myanmar	SEARO	Asia Southeast	South-eastern Asia	931206	26.3	12.4	24.5	(16.8 to 36.1)	11.4	(24.5 to 35.1)	11.7	(24.5 to 35.1)	1.5	(1.1 to 5.6)	115500	228200	(156000 to 336400)	106000	(69100 to 159700)	108600	(70700 to 164700)	13600	(4700 to 34800)
NAM	Namibia	AFRO	Sub-Saharan Africa Southern	Sub-Saharan Africa	58821	18.2	14.4	16.1	(9.4 to 26.9)	8.0	(16.8 to 26.8)	6.8	(16.8 to 26.8)	1.2	(0.9 to 4.6)	8500	9500	(5500 to 15800)	4700	(2500 to 8500)	4000	(2000 to 7200)	700	(200 to 1800)
NPL	Nepal	SEARO	Asia South	Southern Asia	575020	24.2	14.0	30.1	(20.5 to 43.6)	14.1	(31.9 to 41.3)	14.4	(31.9 to 41.3)	1.7	(1.9 to 4.3)	80500	173100	(117800 to 250700)	80800	(52900 to 119200)	82800	(54100 to 122400)	9500	(4400 to 19700)
NIC	Nicaragua	AMRO	Latin America Central	Latin America and the Caribbean	141557	11.9	9.3	9.8	(8.4 to 21.4)	5.5	(9.1 to 16.6)	3.2	(9.1 to 16.6)	1.1	(1.1 to 4.8)	13200	13900	(11900 to 30300)	7700	(6200 to 17200)	4500	(3700 to 10200)	1600	(900 to 5200)
NER	Niger	AFRO	Sub-Saharan Africa West	Sub-Saharan Africa	827124	28.2	9.4	25.3	(14.2 to 43)	13.2	(30 to 42.6)	11.2	(30 to 42.6)	0.8	(0.8 to 4.1)	77700	208900	(117100 to 355500)	109500	(56400 to 195600)	92900	(47700 to 170100)	6600	(2500 to 21000)

NGA	Nigeria	AFRO	Sub-Saharan Africa West	Sub-Saharan Africa	6800000	39.2	12.2	15.6 (9.1 to 26.3)	7.9 (18.5 to 25.1)	6.7 (18.5 to 25.1)	1.0 (1.2 to 2.8)	831100	1061900 (622200 to 1790000)	536400 (291300 to 975700)	455100 (237200 to 811800)	70300 (30400 to 145900)
OMN	Oman	EMRO	North Africa/Middle East	Western Asia	75414	6.7	14.3	14.8 (9.5 to 23.1)	6.5 (11.9 to 22.9)	6.7 (11.9 to 22.9)	1.7 (1.7 to 4.8)	10800	11200 (7100 to 17400)	4900 (2900 to 8000)	5000 (3000 to 8200)	1300 (500 to 2800)
PAK	Pakistan	EMRO	Asia South	Southern Asia	4800000	42.2	15.8	36.0 (24.8 to 52.3)	16.9 (39.4 to 49.9)	17.3 (39.4 to 49.9)	1.9 (2.2 to 5)	757900	1729200 (1190200 to 2508800	810100 (528900 to 1193700)	829800 (544000 to 1230100)	89300 (40600 to 186400)
PAN	Panama	AMRO	Latin America Central	Latin America and the Caribbean	74857	8.5	8.1	8.9 (7.4 to 19.2)	5.0 (8.1 to 15.5)	2.9 (8.1 to 15.5)	1.0 (1.1 to 2.9)	6100	6600 (5600 to 14400)	3700 (2900 to 8300)	2200 (1700 to 5000)	700 (500 to 1700)
PNG	Papua New Guinea	WPRO	Oceana	Oceania	208751	24.3	6.5	16.6 (10.8 to 25.9)	7.8 (15.2 to 26.1)	8.0 (15.2 to 26.1)	0.8 (0.7 to 3.7)	13600	34700 (22500 to 54000)	16300 (10000 to 25800)	16700 (10600 to 26600)	1600 (600 to 5100)
PRY	Paraguay	AMRO	Latin America Tropical	Latin America and the Caribbean	156881	12.4	7.8	7.3 (6.1 to 16.1)	4.0 (6.3 to 12.8)	2.3 (6.3 to 12.8)	0.9 (0.9 to 4.4)	12200	11400 (9500 to 25300)	6300 (4800 to 14400)	3700 (2800 to 8500)	1500 (800 to 4900)
PER	Peru	AMRO	Latin America Andean	Latin America and the Caribbean	594722	9.3	7.3	7.7 (6.3 to 16.7)	4.3 (6.9 to 13.5)	2.5 (6.9 to 13.5)	0.9 (0.9 to 2.4)	43400	45600 (37200 to 99400)	25400 (19400 to 58000)	14900 (11500 to 34500)	5200 (3100 to 11600)
PHL	Philippines	WPRO	Asia Southeast	South-eastern Asia	2300000	14	14.9	25.5 (17.2 to 37.9)	11.7 (25.5 to 36.7)	12.0 (25.5 to 36.7)	1.8 (1.4 to 6.8)	343400	588400 (395600 to 871500)	270700 (172000 to 404800)	277300 (176400 to 419700)	40500 (14700 to 104300)
QAT	Qatar	EMRO	North Africa/Middle East	Western Asia	21528	3.9	10.5	11.0 (6.8 to 18.1)	4.8 (8 to 18.5)	4.9 (8 to 18.5)	1.3 (0.9 to 4.9)	2300	2400 (1500 to 3900)	1000 (600 to 1800)	1100 (600 to 1800)	300 (97 to 700)
KOR	Republic of Korea	WPRO	Asia East	Eastern Asia	509453	1.6	9.2	7.3 (4.1 to 12.7)	3.1 (4.4 to 13.4)	3.1 (4.4 to 13.4)	1.1 (1.1 to 3.9)	46900	37200 (20900 to 64800)	15600 (7900 to 29200)	16000 (8100 to 29700)	5500 (2200 to 12700)
RWA	Rwanda	AFRO	Sub-Saharan Africa East	Sub-Saharan Africa	443228	20.9	9.5	8.0 (4.5 to 14.3)	3.9 (8.1 to 14.2)	3.3 (8.1 to 14.2)	0.8 (0.6 to 3.6)	42100	35500 (20200 to 63600)	17300 (8800 to 32100)	14700 (7400 to 27800)	3600 (1200 to 10200)
LCA	Saint Lucia	AMRO	Caribbean	Latin America and the Caribbean	2862	10.4	11.1	10.4 (8.8 to 22.9)	5.8 (9.7 to 17.7)	3.4 (9.7 to 17.7)	1.2 (1.2 to 4.5)	300	300 (300 to 700)	200 (100 to 400)	98 (78 to 200)	33 (21 to 100)
VCT	Saint Vincent and the Grenadines	AMRO	Caribbean	Latin America and the Caribbean	1861	14.6	11.8	9.0 (7.7 to 19.9)	4.9 (8.1 to 15)	2.9 (8.1 to 15)	1.3 (1.3 to 5.2)	200	200 (100 to 400)	91 (72 to 200)	53 (42 to 100)	23 (15 to 72)
WSM	Samoa	WPRO	Oceana	Oceania	5153	7.2	5.5	2.5 (1.2 to 5.5)	1.0 (1 to 5.7)	1.0 (1 to 5.7)	0.5 (0.5 to 2.8)	300	100 (63 to 300)	49 (18 to 100)	50 (18 to 100)	27 (12 to 93)
STP	Sao Tome and Principe	AFRO	Sub-Saharan Africa West	Sub-Saharan Africa	6421	19.9	10.5	10.2 (6 to 17.5)	5.1 (11 to 17.4)	4.3 (11 to 17.4)	0.8 (0.7 to 3.5)	700	700 (400 to 1100)	300 (200 to 600)	300 (100 to 500)	52 (17 to 100)
SAU	Saudi Arabia	EMRO	North Africa/Middle East	Western Asia	559323	5.2	6.0	11.1 (6.7 to 18.5)	5.2 (8.8 to 19.4)	5.3 (8.8 to 19.4)	0.7 (0.8 to 1.8)	33600	62300 (37400 to 103600)	28800 (16400 to 49300)	29500 (16800 to 50300)	4000 (1900 to 7600)
SEN	Senegal	AFRO	Sub-Saharan Africa West	Sub-Saharan Africa	511506	24.4	9.7	19.2 (11 to 32.4)	9.9 (22.2 to 31.9)	8.4 (22.2 to 31.9)	0.8 (0.7 to 3.9)	49600	98100 (56300 to 165700)	50800 (26300 to 90400)	43100 (22200 to 78500)	4200 (1400 to 12700)
SYC	Seychelles	AFRO	Asia Southeast	Sub-Saharan Africa	1471	8.3	11.6	10.0 (5.8 to 17.9)	5.0 (9.7 to 18.2)	4.3 (9.7 to 18.2)	0.8 (0.8 to 4)	200	100 (85 to 300)	74 (37 to 100)	63 (31 to 100)	11 (5 to 39)
SLE	Sierra Leone	AFRO	Sub-Saharan Africa West	Sub-Saharan Africa	211899	49.5	10.0	18.7 (11.1 to 32.3)	9.7 (22.7 to 30.8)	8.2 (22.7 to 30.8)	0.8 (0.6 to 3.3)	21200	39700 (23500 to 68500)	20500 (11100 to 36900)	17400 (9100 to 31600)	1800 (600 to 5000)
SGP	Singapore	WPRO	Asia Pacific High Income	South-eastern Asia	55968	1.2	11.5	13.3 (8.2 to 21.3)	5.9 (10.4 to 21.5)	6.0 (10.4 to 21.5)	1.4 (1.1 to 5.4)	6400	7400 (4600 to 11900)	3300 (1900 to 5500)	3400 (2000 to 5600)	800 (300 to 1900)
SLB	Solomon Islands	WPRO	Oceana	Oceania	17028	13.8	12.4	18.2 (11.9 to 27.5)	8.3 (16.3 to 27.3)	8.5 (16.3 to 27.3)	1.4 (1.2 to 6)	2100	3100 (2000 to 4700)	1400 (900 to 2200)	1400 (900 to 2300)	200 (89 to 700)
SOM	Somalia	EMRO	Sub-Saharan Africa East	Sub-Saharan Africa	444651	45.7	12.0	16.5 (9.6 to 28.1)	8.4 (19.2 to 27.1)	7.1 (19.2 to 27.1)	1.0 (0.8 to 3.8)	53400	73300 (42500 to 124800)	37200 (20300 to 67900)	31600 (16500 to 57700)	4500 (1600 to 12200)
ZAF	South Africa	AFRO	Sub-Saharan Africa Southern	Sub-Saharan Africa	1100000	15.3	8.0	15.2 (8.5 to 26.5)	7.9 (16 to 25.2)	6.7 (16 to 25.2)	0.7 (0.7 to 2.7)	88300	168000 (93300 to 291200)	86800 (45200 to 155200)	73700 (37400 to 131700)	7500 (2800 to 19100)
LKA	Sri Lanka	SEARO	Asia Southeast	Southern Asia	382027	6.1	10.7	18.5 (12.1 to 27.9)	8.5 (16.7 to 28.1)	8.7 (16.7 to 28.1)	1.3 (1.4 to 4.2)	40900	70500 (46100 to 106600)	32500 (19900 to 50000)	33200 (20800 to 51900)	4800 (2100 to 11100)
SDN	Sudan	EMRO	Sub-Saharan Africa East	Sub-Saharan Africa	1200000	28.6	13.2	27.5 (15.8 to 47.2)	14.3 (32.8 to 47.4)	12.1 (32.8 to 47.4)	1.1 (1.2 to 3.4)	161200	336300 (190000 to 566600)	174600 (89700 to 311200)	148100 (75500 to 272100)	13600 (5200 to 28100)
SUR	Suriname	AMRO	Caribbean	Latin America and the Caribbean	9437	11.7	8.8	11.7 (10.2 to 24.5)	6.7 (11.5 to 19.6)	3.9 (11.5 to 19.6)	1.1 (1.2 to 3.3)	800	1100 (1000 to 2300)	600 (500 to 1400)	400 (300 to 800)	100 (61 to 300)
SWZ	Swaziland	AFRO	Sub-Saharan Africa Southern	Sub-Saharan Africa	36777	29.5	13.9	12.5 (7.5 to 21)	6.1 (13.9 to 20.4)	5.2 (13.9 to 20.4)	1.2 (1 to 4.6)	5100	4600 (2800 to 7700)	2300 (1200 to 4100)	1900 (1000 to 3400)	400 (100 to 1200)
SYR	Syrian Arab Republic	EMRO	North Africa/Middle East	Western Asia	535264	8.5	10.9	14.3 (9.2 to 22.6)	6.5 (11.9 to 22.8)	6.6 (11.9 to 22.8)	1.3 (1 to 5.3)	58300	76800 (49400 to 121000)	34500 (20400 to 56200)	35400 (21300 to 57700)	6900 (2300 to 18000)
TJK	Tajikistan	EURO	Asia Central	Caucasus and Central Asia	269759	23	10.7	12.8 (7.9 to 20.4)	5.7 (10 to 21)	5.8 (10 to 21)	1.3 (1 to 4.8)	28900	34500 (21200 to 55200)	15400 (8800 to 25400)	15700 (9200 to 26400)	3400 (1200 to 9200)
THA	Thailand	SEARO	Asia Southeast	South-eastern Asia	698934	8.1	12.0	14.1 (9 to 22.3)	6.3 (11.4 to 22.3)	6.4 (11.4 to 22.3)	1.4 (1.8 to 3.7)	83900	98600 (63100 to 155600)	43800 (25800 to 71900)	44900 (26800 to 73500)	9900 (4800 to 20500)
TLS	Timor-Leste	SEARO	Asia Southeast	South-eastern Asia	42075	24.4	12.1	20.0 (13.4 to 29.9)	9.2 (18.7 to 29.6)	9.4 (18.7 to 29.6)	1.4 (1.2 to 5.9)	5100	8400 (5700 to 12600)	3900 (2400 to 5900)	4000 (2500 to 6200)	600 (200 to 1600)
TGO	Togo	AFRO	Sub-Saharan Africa West	Sub-Saharan Africa	238470	32.6	13.3	14.9 (8.7 to 25.1)	7.5 (17.3 to 23.9)	6.3 (17.3 to 23.9)	1.1 (0.9 to 4.6)	31700	35600 (20700 to 59800)	17800 (9600 to 32200)	15100 (7900 to 26900)	2700 (900 to 7200)
TON	Tonga	WPRO	Oceana	Oceania	2703	6.6	7.5	5.5 (3.1 to 10.2)	2.3 (3 to 10.9)	2.4 (3 to 10.9)	0.8 (0.7 to 3.4)	200	100 (83 to 300)	62 (29 to 100)	64 (30 to 100)	23 (9 to 66)
TTO	Trinidad and Tobago	AMRO	Caribbean	Latin America and the Caribbean	19543	14.8	8.1	11.2 (9.8 to 24)	6.5 (11.1 to 19)	3.8 (11.1 to 19)	1.0 (1.1 to 3)	1600	2200 (1900 to 4700)	1300 (1000 to 2800)	700 (600 to 1600)	200 (100 to 500)
TUN	Tunisia	EMRO	North Africa/Middle East	Northern Africa	190386	9.5	8.9	6.2 (3.5 to 11.2)	2.9 (5.4 to 11.7)	2.5 (5.4 to 11.7)	0.7 (0.7 to 2.6)	16900	11800 (6600 to 21300)	5600 (2800 to 10900)	4800 (2300 to 9400)	1400 (600 to 3200)
TUR	Turkey	EURO	North Africa/Middle East	Western Asia	1300000	8.5	12.0	16.4 (10.4 to 25)	7.4 (14.1 to 25.1)	7.6 (14.1 to 25.1)	1.4 (1.6 to 4.1)	154500	210600 (135500 to 325600)	95000 (57100 to 150400)	97300 (59800 to 154000)	18200 (8600 to 37900)
TKM	Turkmenistan	EURO	Asia Central	Caucasus and Central Asia	103913	21.9	9.8	10.0 (6.1 to 16.7)	4.4 (7 to 17.4)	4.5 (7 to 17.4)	1.2 (0.9 to 4.6)	10200	10400 (6300 to 17300)	4600 (2500 to 7900)	4700 (2500 to 8200)	1200 (400 to 3100)
UGA	Uganda	AFRO	Sub-Saharan Africa East	Sub-Saharan Africa	1500000	22.6	13.6	16.1 (9.6 to 27.1)	8.1 (18.4 to 26.2)	6.8 (18.4 to 26.2)	1.1 (1.2 to 4.2)	208100	245700 (144400 to 407200)	123400 (64700 to 225200)	104700 (54300 to 187100)	17500 (6600 to 41300)
ARE	United Arab Emirates	EMRO	North Africa/Middle East	Western Asia	143597	5	7.6	5.6 (3.1 to 10.1)	2.3 (3.1 to 11)	2.4 (3.1 to 11)	0.9 (1 to 2.4)	10900	8100 (4400 to 14500)	3400 (1600 to 6700)	3400 (1600 to 6800)	1300 (500 to 2700)
TZA	United Republic of Tanzania	AFRO	Sub-Saharan Africa East	Sub-Saharan Africa	1800000	21.4	11.4	11.6 (7.1 to 20.1)	5.8 (12.5 to 19)	4.9 (12.5 to 19)	1.0 (0.7 to 3.8)	210300	214400 (128000 to 362400)	106300 (55900 to 189800)	90200 (46600 to 163100)	17800 (5600 to 49300)
URY	Uruguay	AMRO	Latin America Southern	Latin America and the Caribbean	49186	3.9	10.1	9.4 (8 to 20.5)	5.2 (8.4 to 16.1)	3.1 (8.4 to 16.1)	1.2 (1.9 to 2.4)	5000	4600 (3900 to 10100)	2600 (2000 to 5700)	1500 (1200 to 3400)	600 (400 to 1100)
UZB	Uzbekistan	EURO	Asia Central	Caucasus and Central Asia	631538	13.5	8.7	9.1 (5.5 to 15.3)	4.0 (6.3 to 16.2)	4.1 (6.3 to 16.2)	1.0 (1 to 3.7)	54900	57600 (34600 to 96900)	25200 (13300 to 44800)	25900 (13800 to 45600)	6500 (2700 to 14600)
VUT	Vanuatu	WPRO	Oceana	Oceania	7087	8.8	12.9	15.2 (9.8 to 23.6)	6.8 (12.5 to 23.6)	6.9 (12.5 to 23.6)	1.5 (1.2 to 6.2)	900	1100 (700 to 1700)	500 (300 to 800)	500 (300 to 800)	100 (36 to 300)
VEN	Venezuela	AMRO	Latin America Central	Latin America and the Caribbean	598602	8.5	9.0	8.7 (8.8 to 20.9)	4.8 (7.8 to 15)	2.8 (7.8 to 15)	1.1 (2.6 to 6.5)	48500	52100 (52500 to 125000)	28800 (22300 to 64700)	16900 (13300 to 38600)	6400 (8800 to 31100)
VNM	Viet Nam	WPRO	Asia Southeast	South-eastern Asia	1400000	12.4	9.4	9.4 (5.8 to 15.6)	4.1 (6.4 to 16)	4.2 (6.4 to 16)	1.1 (0.8 to 4.2)	132400	131900 (81000 to 217700)	57400 (30900 to 101200)	58800 (31600 to 102500)	15700 (5200 to 43500)
YEM	Yemen	EMRO	North Africa/Middle East	Western Asia	726617	27	13.2	32.5 (22.4 to 47.2)	15.3 (35.1 to 44.8)	15.7 (35.1 to 44.8)	1.6 (1.1 to 6.1)	95900	236400 (162500 to 343000)	111200 (72800 to 164100)	113900 (74300 to 168400)	11300 (3900 to 28000)
ZMB	Zambia	AFRO	Sub-Saharan Africa East	Sub-Saharan Africa	582213	29.4	12.9	13.4 (7.8 to 22.5)	6.7 (15 to 21.9)	5.6 (15 to 21.9)	1.1 (0.8 to 4.5)	75100	78000 (45400 to 131200)	38700 (20700 to 69500)	32900 (16900 to 59000)	6400 (1900 to 18400)
ZWE	Zimbabwe	AFRO	Sub-Saharan Africa Southern	Sub-Saharan Africa	437601	38.8	16.6	14.3 (8.4 to 24)	7.0 (15.8 to 22.8)	5.9 (15.8 to 22.8)	1.4 (1.7 to 3.7)	72600	62500 (36800 to 104900)	30500 (16400 to 54900)	25900 (13300 to 46400)	6100 (2700 to 12400)

SOURCES
* Live births: SOWC 2012; http://www.unicef.org/sowc2012/statistics.php
** UN IGME: http://www.childmortality.org/
*** Born too Soon Report: Howson, C.P.; Kinney, M.V.; Lawn, J.E. (Editors). Born too soon: the global action report on preterm birth. World Health Organization, Geneva, Switzerland (2012) 126 pp. ISBN 978 92 4 150343 3
