# Supplementary material for: Estimates of burden and consequences of infants born small for gestational age in low and middle income countries with INTERGROWTH-21st standard: analysis of CHERG datasets
Source: BMJ. 2017 Aug 17;358:j3677. doi: 10.1136/bmj.j3677 (PMC5558898; doi:10.1136/bmj.j3677)
Supplement: Supplementary file 6 — Appendix 6: Numbers of small for gestational age infants born in 2012 comparing INTERGROWTH-21st birth weight standard with US 1991 birth weight reference [file leea038389.ww6.pdf]

Appendix 6: Numbers of SGA Infants born in 2012 comparing the INTERGROWTH-21st birth weight standard to the U.S. 1991 birth weight reference [posted as supplied by author]

		Intergrowth 21st Standard					U.S. 1991 Reference				
**UN-MDG Region**	**Live Births, n***	**Term-SGA-non-LBW Births, n**	**Term-SGA-LBW Births, n**	**Preterm-SGA Births, n**	**Total SGA Births, n**	**Total SGA Prevalence (%)**	**Term-SGA-non-LBW Births, n**	**Term-SGA-LBW Births, n**	**Preterm-SGA Births, n**	**Total SGA Births, n**	**Total SGA Prevalence (%)**
Caucasus / Central Asia	1,774,300	87,000	89,100	19,400	195,500	11.0	134,400	89,600	36,700	260,700	14.7
Eastern Asia	19,097,200	387,400	396,800	165,400	949,500	5.0	598,300	398,900	314,000	1,311,200	6.9
Latin America / Caribbean	10,833,300	516,300	303,200	110,800	930,300	8.6	842,300	344,000	192,800	1,379,100	12.7
Northern Africa	3,989,800	120,900	102,600	24,600	248,200	6.2	247,600	87,000	47,000	381,500	9.6
Oceania	266,400	20,000	20,400	2,300	42,700	16.0	30,800	20,500	4,400	55,700	20.9
South-eastern Asia	9,691,100	941,700	964,600	183,500	2,089,900	21.6	1,454,600	969,700	347,200	2,771,500	28.6
Southern Asia	36,625,800	5,908,500	6,052,100	577,100	12,537,700	34.2	9,126,200	6,084,200	1,094,100	16,304,400	44.5
Sub-Saharan Africa	33,727,500	2,829,500	2,400,600	345,000	5,575,200	16.5	5,792,600	2,035,300	655,800	8,483,700	25.2
Western Asia	4,844,900	346,000	354,400	56,200	756,600	15.6	534,400	356,300	106,200	996,900	20.6
**LMIC TOTAL**	**120,850,200**	**11,157,400**	**10,683,900**	**1,484,300**	**23,325,600**	**19.3**	**18,761,400**	**10,385,500**	**2,797,900**	**31,944,800**	**26.4**

*All numerical estimates were rounded to the nearest 100s.

Abbreviations: SGA = Small-for-gestational-age; LBW = Low birth weight (<2500g); LMIC= Low- and middle-income countries; UN-MDG = United Nations Millennium Development Goals
