# Supplementary material for: Estimates of burden and consequences of infants born small for gestational age in low and middle income countries with INTERGROWTH-21st standard: analysis of CHERG datasets
Source: BMJ. 2017 Aug 17;358:j3677. doi: 10.1136/bmj.j3677 (PMC5558898; doi:10.1136/bmj.j3677)
Supplement: Supplementary file 8 — Appendix 8: Neonatal deaths in 2012 attributable to term-small for gestational age and preterm-small for gestational age in low and middle income countries comparing INTERGROWTH-21st birth weight standard to US 1991 birth weight reference [file leea038389.ww8.pdf]

Appendix 8: Neonatal Deaths in 2012 Attributable to Term-SGA and Preterm-SGA in LMICs comparing the INTERGROWTH-21st birth weight standard to the U.S. 1991 birth weight reference [posted as supplied by author]

			Intergrowth 21st Standard					U.S. 1991 Reference				
**UN-MDG Region**	**Live Births, n***	**Neonatal Deaths, n**	**Term-SGA-not-LBW Neonatal Deaths, n** (UR**)	**Term-SGA-LBW Neonatal Deaths, n** (UR**)	**Preterm-SGA Neonatal Deaths, n** (UR**)	**All SGA Neonatal Deaths, n** (UR**)	**% of neonatal deaths attributable to SGA** (UR**)	**Term-SGA-not-LBW Neonatal Deaths, n** (UR**)	**Term-SGA-LBW Neonatal Deaths, n** (UR**)	**Preterm-SGA Neonatal Deaths, n** (UR**)	**All SGA Neonatal Deaths, n** (UR**)	**Population Attributable Fraction: % of neonatal deaths attributable to SGA** (UR**)
Caucasus / Central Asia	1,774,300	26,500	-	1,500	2,300	3,800	14.3	-	1400	4,000	5,500	20.6
Eastern Asia	19,097,200	158,900	-	4,200	11,900	16,100	10.1	-	3900	21,200	25,100	15.8
Latin America / Caribbean	10,833,300	105,900	-	8,600	18,700	27,300	25.8	-	8600	28,500	37,100	35.0
Northern Africa	3,989,800	50,600	1,600	2,700	900	5,200	10.3	3100	2,215	1,600	7,000	13.8
Oceania	266,400	5,700	-	500	400	900	15.8	-	500	700	1,200	20.6
South-eastern Asia	9,691,100	143,900	-	14,500	18,500	33,000	22.9	-	13,300	31,800	45,100	31.3
Southern Asia	36,625,800	1,127,300	-	175,800	113,900	289,700	25.7	-	162,100	197,200	359,300	31.9
Sub-Saharan Africa	33,727,500	1,090,200	72,200	123,200	23,900	219,300	20.1	137,500	97,200	42,400	277,100	25.4
Western Asia	4,844,900	63,400	-	5,600	5,400	11,000	17.4	-	5,200	9,500	14,700	23.2
**LMIC TOTAL**	**120,850,200**	**2,772,400**	**73,800**	**336,800**	**195,900**	**606,500**	**21.9**	**140,600**	**294,500**	**336,900**	**772,000**	**27.8**

*All numerical estimates were rounded to the nearest 100s.

**Uncertainty ranges (UR) were derived with a bootstrap approach (appendix 4).

Abbreviations: SGA= Small-for-gestational-age; LMICs= Low- and middle-income countries; UR= uncertainty range; LBW= low birth weight (<2500g); UN-MDG=United Nations Millennium Development Goals
