# Supplementary material for: Estimates of burden and consequences of infants born small for gestational age in low and middle income countries with INTERGROWTH-21st standard: analysis of CHERG datasets
Source: BMJ. 2017 Aug 17;358:j3677. doi: 10.1136/bmj.j3677 (PMC5558898; doi:10.1136/bmj.j3677)
Supplement: Supplementary file 9 — Appendix 9: Neonatal deaths averted by reducing small for gestational age prevalence to 10% in all low and middle income countries in 2012 [file leea038389.ww9.pdf]

Appendix 9: Neonatal Deaths Averted by Reducing SGA Prevalence to 10% in All LMICs in 2012 [posted as supplied by author]

			Intergrowth 21st Standard			
**UN-MDG Region**	**Live Births 2012, n***	**Neonatal Deaths 2012, n**	**Term-SGA-not-LBW, n (UR**)**	**Term-SGA-LBW, n (UR**)**	**Preterm-SGA, n (UR**)**	**All SGA, n (UR**)**
Caucasus / Central Asia	1,774,300	26,500	-	200 (0 to 1,200)	200 (0 to 2,100)	400 (0 to 2,600)
Eastern Asia	19,097,200	158,900	-	100 (0 to 300)	100 (0 to 600)	100 (0 to 700)
Latin America / Caribbean	10,833,300	105,900	-	600 (400 to 8,300)	300 (100 to 13,600)	1,000 (800 to 18,000)
Northern Africa	3,989,800	50,600	0 (0 to 400)	0 (0 to 700)	0 (0 to 300)	0 (0 to 1,400)
Oceania	266,400	5,700	-	200 (0 to 500)	100 (0 to 400)	300 (100 to 700)
South-eastern Asia	9,691,100	143,900	-	7,400 (2,400 to 14,400)	2,600 (100 to 18,400)	9,900 (4,400 to 26,500)
Southern Asia	36,625,800	1,127,300	-	130,300 (75,000 to 203,100)	17,100 (400 to 115,100)	147,400 (94,100 to 253,900)
Sub-Saharan Africa	33,727,500	1,090,200	31,700 (4,200 to 75,800)	54,200 (6,600 to 128,300)	6,100 (500 to 22,400)	92,000 (15,600 to 215,100)
Western Asia	4,844,900	63,400	-	2,600 (1,100 to 5,500)	800 (0 to 5,300)	3,400 (1,700 to 8,100)
**LMIC TOTAL*** **	**120,850,200**	**2,772,400**	**31,700 (3,800 to 76,100)**	**195,600 (114,600 to 311,200)**	**27,200 (7,700 to 157,300)**	**254,600 (164,800 to 449,700)**

*All rounded to the nearest 100s.
**Uncertainty ranges (UR) were derived with a bootstrap approach (appendix 4).
***The LMIC total numbers, rounded after summing national estimates, may not equal the sum of rounded regional estimates presented here.
Abbreviations: SGA= Small-for-gestational-age; LMICs= Low- and middle-income countries; UR= uncertainty range; UN-MDG= United Nations Millennium Development Goals
